# Web 2.0-Based Crowdsourcing for High-Quality Gold Standard Development in Clinical Natural Language Processing

**DOI:** 10.2196/jmir.2426

**Published:** 2013-04-02

**Authors:** Haijun Zhai, Todd Lingren, Louise Deleger, Qi Li, Megan Kaiser, Laura Stoutenborough, Imre Solti

**Affiliations:** ^1^Division of Biomedical Informatics, Cincinnati Children’s Hospital Medical CenterCincinnati, OHUnited States

**Keywords:** clinical informatics, natural language processing, named entity, reference standards, crowdsourcing, user computer interface, quality control

## Abstract

**Background:**

A high-quality gold standard is vital for supervised, machine learning-based, clinical natural language processing (NLP) systems. In clinical NLP projects, expert annotators traditionally create the gold standard. However, traditional annotation is expensive and time-consuming. To reduce the cost of annotation, general NLP projects have turned to crowdsourcing based on Web 2.0 technology, which involves submitting smaller subtasks to a coordinated marketplace of workers on the Internet. Many studies have been conducted in the area of crowdsourcing, but only a few have focused on tasks in the general NLP field and only a handful in the biomedical domain, usually based upon very small pilot sample sizes. In addition, the quality of the crowdsourced biomedical NLP corpora were never exceptional when compared to traditionally-developed gold standards. The previously reported results on medical named entity annotation task showed a 0.68 F-measure based agreement between crowdsourced and traditionally-developed corpora.

**Objective:**

Building upon previous work from the general crowdsourcing research, this study investigated the usability of crowdsourcing in the clinical NLP domain with special emphasis on achieving high agreement between crowdsourced and traditionally-developed corpora.

**Methods:**

To build the gold standard for evaluating the crowdsourcing workers’ performance, 1042 clinical trial announcements (CTAs) from the ClinicalTrials.gov website were randomly selected and double annotated for medication names, medication types, and linked attributes. For the experiments, we used CrowdFlower, an Amazon Mechanical Turk-based crowdsourcing platform. We calculated sensitivity, precision, and F-measure to evaluate the quality of the crowd’s work and tested the statistical significance (*P*<.001, chi-square test) to detect differences between the crowdsourced and traditionally-developed annotations.

**Results:**

The agreement between the crowd’s annotations and the traditionally-generated corpora was high for: (1) annotations (0.87, F-measure for medication names; 0.73, medication types), (2) correction of previous annotations (0.90, medication names; 0.76, medication types), and excellent for (3) linking medications with their attributes (0.96). Simple voting provided the best judgment aggregation approach. There was no statistically significant difference between the crowd and traditionally-generated corpora. Our results showed a 27.9% improvement over previously reported results on medication named entity annotation task.

**Conclusions:**

This study offers three contributions. First, we proved that crowdsourcing is a feasible, inexpensive, fast, and practical approach to collect high-quality annotations for clinical text (when protected health information was excluded). We believe that well-designed user interfaces and rigorous quality control strategy for entity annotation and linking were critical to the success of this work. Second, as a further contribution to the Internet-based crowdsourcing field, we will publicly release the JavaScript and CrowdFlower Markup Language infrastructure code that is necessary to utilize CrowdFlower’s quality control and crowdsourcing interfaces for named entity annotations. Finally, to spur future research, we will release the CTA annotations that were generated by traditional and crowdsourced approaches.

## Introduction

One of the key components of supervised machine learning-based clinical natural language processing (NLP) systems is the high-quality gold standard used for training and testing. In clinical NLP projects, expert annotators are traditionally asked to double annotate the text for the purposes of the gold standard. Expert annotators could be clinicians or extensively trained laypeople [1]. Unless the expert annotators are volunteers, they are very costly to pay and it is usually not easy to build a sufficiently large group of expert annotators locally and, consequently, fast contingent of annotators. To reduce the cost of expert human annotation, many projects in general NLP have turned to crowdsourcing, which involves submitting a large number of smaller subtasks to a coordinated marketplace of workers on the Internet. These workers (called turkers) are paid small amounts (usually a few cents) for each task, sometimes resulting in considerable overall savings over the traditional expert annotator model. The trade-off is usually between the accuracy of the annotation result and the cost savings. Because anonymous turkers from all over the world have different levels of proficiency in the task and are not trained to accomplish the task, efficient quality control and judgment voting methods are required to generate good results.

Many studies have been conducted in the area of crowdsourcing tasks. As early as 2008, Snow et al [2] were the first to explore the feasibility of crowdsourcing in NLP. Five NLP tasks were published on Amazon Mechanical Turk (AMT, [3]) to turkers. Their results indicated that non-expert labellers could obtain high-quality annotations. Since then, data created by crowdsourcing has been widely studied for different research areas. Lawson et al [4] described how using a competitive payment system and interannotator agreement improved the quality of named entity annotations on AMT. Unlike traditional named entity experiments, Finin et al [5] presented their experience by leveraging AMT and CrowdFlower [6] to annotate named entities in Twitter data. It was the first work of named entity recognition in the new domains of Facebook and Twitter. Meanwhile, several studies attempted to use crowdsourcing to create data for machine translation systems. Ambati and Vogel [7] explored the effectiveness of using AMT to do sentence translation for creating parallel corpora. Denkowski et al [8-10] attempted to generate annotated data in a variety of languages. In addition, crowdsourcing was also applied to transcription [11-13], part-of-speech tagging [14], and other tasks [15,16].

In a recent publication of the Journal of Medical Internet Research, Turner et al [17] reported on the use of crowdsourcing to collect feedback on the design of health promotion messages for oral health. Luengo-Oroz et al [18] evaluated the feasibility of crowdsourcing to conduct malaria image analysis. Gathering a large number of high quality annotations is a critical challenge in biomedical NLP, which was presented in detail in the editorial of Chapman et al [1]. As demonstrated by studies in the general NLP field, crowdsourcing is a decidedly promising solution to this research area. However, in contrast to the general NLP domain, there are only a few studies involving crowdsourcing in biomedical NLP and almost none for clinical NLP. Most recently, Burger et al [19] performed a task of extracting the gene-mutation relations in Medical Literature Analysis and Retrieval System Online (MEDLINE) abstracts on AMT. In their work, candidate mutations were extracted from 250 MEDLINE abstracts using the Extractor of Mutations (EMU) presented together with the curated gene lists from the National Center for Biotechnology Information (NCBI). Using a customized interface, it was feasible for turkers to apply their judgments. They reported that the weighted accuracy was 82%. This work was somewhat similar to our linking of medications and their attributes, but it focused on a very specific gene-mutation domain. Norman et al [20] investigated leveraging crowdsourcing to facilitate the discovery of new medicines.

Yetisgen-Yildiz et al [21,22] explored the task of using AMT to annotate biomedical text. Clinical trial announcements annotated 3 entity types (medical conditions, medications, and laboratory tests). The authors indicated that AMT was a very promising tool for annotating clinical text and a well-designed interface and annotation guidelines were helpful to further improve the performance. Building on these earlier works, we designed our medical named entity annotation experiment to include a large-scale data set, easy-to-use graphical user interface and strict quality control. Specifically, a corpus ten times of earlier works was used in our experiments. CrowdFlower Markup Language (CML) and JavaScript were leveraged to implement the interface. We implemented a 4-component quality control strategy to improve the crowd-generated annotation.

Improving the quality of judgments is one of the most important issues in crowdsourcing, especially for the tasks without strong quality control. A variety of methods have been proposed to assess the quality of judgments from turkers. Kumar and Lease [23,24] presented a weighted voting method based on turkers’ accuracies, which can be estimated by taking the full set of labels into account. Jung and Lease [25] conducted a large-scale consensus study on relevant judgements between query/document pairs for Web search on the ClueWeb09 dataset [26]. In their work, approximately 20,000 labels were collected from 766 Mechanical Turk workers. They reported that computing the Z-score could filter noisy labels and achieve a significant improvement, in comparison to a majority vote baseline. Based on the previous work, a semi-supervised approach was proposed to maximize the benefit from consensus [27] with consensus labels from both labelled and unlabeled examples. As these studies indicated, though much progress has been made, quality control and aggregating judgments are still the major challenges of crowdsourcing. The highest reported performance of medication name entity annotation from earlier crowdsourcing attempts in the biomedical NLP domain was 0.68 (F-measure) for agreement between traditional and crowd-generated corpora [21,22].

In our research, we applied strict quality control to select qualified turkers and investigated multiple approaches to aggregating judgments. The goals of our study were to build upon previous work from the general crowdsourcing research and to evaluate the usability of crowdsourcing approach in the clinical NLP domain. This will help us automate clinical trial eligibility screening. The clinical NLP tasks that we used for the purpose of evaluation were medical named entity recognition and entity linking in a clinical trial announcement (CTA) corpus. The entities involved were medication names and medication types, as well as their attributes. During our research, we first studied the turkers’ performance to annotate medical named entities on a large-scale data set. Second, we proposed to use crowdsourcing to link named entities and their attributes, in which the entities and attributes were pre-annotated in the text and the crowdsourcing task was to identify entity/attribute pairs that are associated in the text. Third, we attempted to find a new solution to produce a more robust, manually-created gold standard (ie, correction) by investigating whether an iterative model of crowdsourcing tasks can correct errors from previous generations of tasks. Finally, we studied 3 methods to aggregate multiple annotations of the same text to generate a better gold standard.

Our research contributed to the field of clinical NLP by: (1) evaluating the usability of crowdsourcing in the clinical NLP domain, (2) publicly releasing the user interface software that is necessary for crowdsourced, named-entity annotation, and (3) implementing a 4-component quality control strategy to improve the crowd generated annotation, including an introductory quiz to filter the automated scripts, a geographical constraints for turkers, training turkers for the task, and continuous performance monitoring. We will release the annotated corpora in December 2013 when our NIH grant funding concludes.

## Methods

### Definition of Annotated Named Entities and Linkages

This section presents the definitions and examples of medication entities (medication names and medication types) and medication-attribute linkages annotated in this work.

#### Medication Name

Medication names are specific names of drugs, biological substances, and treatments. Some examples of mediation names are ibuprophen, phosphonoacetic acid, vancomycin, and ganciclovir.

#### Medication Type

Medication types refer to classes of drugs (eg, antibiotics, anti-inflammatory drugs, benzodiazapines), types of drug therapy (eg, chemotherapy), and general references to medications (eg, “study drug”, “other drugs”, “medication”).

#### Attribute

Attributes define how much, how often, and in what form medications or medication types are taken. We distinguished between the following categories of attributes (based on the schema of the SHARPn project [28]):

- Date: indicating all dates associated with the medication (eg, start dates, concluding dates)
- Strength: indicating the strength number and units of the prescribed drug
- Dosage: indicating the amount of each medication used by the patient and type of dose it is (eg, high dose, low dose, stable dose)
- Form: indicating the shape or configuration of the medication (eg, tablet, capsule, liquid, injection, infusion)
- Frequency: indicating how often each dose of the medication should be taken
- Duration: indicating how long the patient is expected to take the drug
- Route: indicating route or method of the medication (eg, intravenous, oral, chew, topical)
- Status change: indicating whether the medication is currently being taken or not (eg, active, inactive, hold, incomplete, started, discontinued, increased, decreased, no change)
- Modifier: indicating mentions that could exist under certain circumstances (eg, conditional modifier), develop or alter a mention (eg, course modifier), or generic modifier (eg, conventional)

### Linking

**Figure 1:**
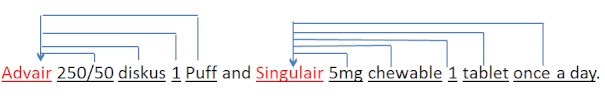
Example of linkages between medications and their attributes.

The linking task associates attributes to their corresponding medication entities, assuming medications and attributes have already been pre-annotated. The following sentence demonstrates the linking task: “Advair 250/50 diskus 1 puff and Singulair 5mg chewable 1 tablet once a day”. In this sentence, “Advair and Singulair” are the medication names and “250/50, diskus, 1, puff, 5mg, chewable, 1, tablet, and once a day” are the attributes. In this example, “250/50, diskus, 1, and puff” are the attributes of Advair, “5mg, chewable, 1, tablet and once a day” are the attributes of Singulair, as shown in Figure 1.

### Gold Standard to Evaluate Turker Performance

In one of our previous projects, CTA were annotated for medication extraction. In this paper, we present the most important features of the gold standard used in the study. Details of the corpora and the process of the gold standard development were thoroughly described in a separate manuscript that was published in the 2012 AMIA Annual Conference Proceedings [29]. The corpus was double annotated for medication names, types, and attributes by two annotators (college graduates with bachelor degrees) to create a gold standard, at a cost of 20 days per annotator for annotation of medication names and types and an additional 20 days per annotator for the attributes. Additionally, each attribute was linked to its respective medication name or medication type.

The CTA corpus was composed of 3000 CTAs randomly selected from the ClinicalTrials website (105,598 documents as of March 2011). We annotated only the eligibility criteria sections of the trial announcements. Table 1 shows the descriptive statistics of the corpus (number of documents and number of annotations in the traditional gold standard). In this study we used crowdsourcing to annotate only medication names and medication types. The linking crowdsourcing experiment utilized pre-annotated text: medication names, medication types, and attributes.

**Table 1:** CTA corpus statistics.

Corpus statistics		
Documents		3000
Tokens		635,003
**Annotations**		
	Medication name	9968
	Medication type	11,789
	Date	16
	Dosage	645
	Duration	644
	Form	482
	Frequency	381
	Route	894
	Status change	598
	Strength	409
	Modifier	5827
	All classes	31,653

Because the CTAs were longer than the text of many crowdsourcing tasks, and considering the difficulty of clinical NLP annotations, we decided to break up the CTAs into smaller paragraph-length sizes for the tasks. Based on a tokenizer we wrote to count discrete basic units, the average token count in a CTA document was 212. In the paragraph-size tasks, we split the CTAs into paragraphs with at least 50 tokens, preserving the original format and the integrity of the CTA file (no paragraphs spanned into different CTAs). This resulted in 9773 paragraphs or “units”.

### Crowdsourcing User Interface

We selected CrowdFlower (CF) as the platform through which we would access AMT because CF’s self-service product met our needs for flexibility in graphical user interface (GUI) modification and offered means for strict and continuous quality control for the annotations. We wrote a custom JavaScript program that was loaded into a CF job, allowing the turkers to highlight and classify entities in a similar fashion to the Knowtator plug-in for Protégé [30] that was used in our traditional annotation methods.

In addition to the customizable GUI, another key benefit of using the CF crowdsourcing platform over directly accessing AMT is that it has strict quality control measures. CF provides an interface for creating and editing “gold standard answers” for quality control. “Gold standard answers” are randomly included (without the turkers being aware of their presence) in the submitted data and a turker is required to meet a minimum threshold of accuracy in these “gold” examples in order to continue submitting tasks. When a turker meets this threshold, he/she is deemed “trusted”. Only the “trusted” turkers’ data are collected to establish final judgments. If an example has 3 medications in the unit and the turker annotates only two correctly, the system will score the judgment as incorrect as there are no partial scores in determining a turker’s trust status within a particular unit of annotated text.

In pilots, we experimented with different thresholds. Lower thresholds resulted in lower agreement of the turker-annotated corpora with the gold standard corpora. Higher thresholds prevented the successful completion of the task by eliminating too many turkers. Because of the complexity of the task, we experimented with a trust-based threshold and found 50% (on unit level) to be the most feasible threshold number. A turker presented with “gold standard” examples must accurately annotate 50% of the unit-based responses. That is, if the turker annotated 4 units of “gold” examples, at least 2 (of the 4) had to be exact matches for him/her to establish trustworthiness. The 50% threshold was evaluated on the unit’s level and not on the named entity level. That is, all of a unit’s annotations, or judgments, had to be matched exactly with the “gold standard” answers, irrespective the number of named entities per unit.

We also found that the training mode of CF was very helpful in winnowing the pool of turker candidates to only the highest quality annotators. In training mode, the turkers were directed to several training examples first. All of the training examples were gold standard examples and the turker must complete 4 examples correctly to proceed to the production annotation task. Based on these interfaces, we implemented our quality control strategy. Of all our tasks, 20% of the total number of units submitted for judgment were uploaded and setup as “gold” units. That is, 20% of the annotated units were gold standard units where the CF system could continuously gauge the trustworthiness of the turker. If a turker’s trustworthiness slipped below 50%, the turker was warned. If his/her performance did not improve during the next two gold tests, then the turker’s entire output was excluded from the collected data and the system subsequently blocked the turker from submitting any further judgements.

In the in-house experts’ generated gold standard, approximately 30% of the CTAs had no medications or medication types. Due to the splitting of the CTAs into smaller units, however, the empty percentage grew to 42%. Several initial pilot experiments were conducted regarding the study’s design features, including training mode, trusted-turker accuracy threshold, and whether empty tasks were included or not. We tested the performance of excluding empty units (where the data included at least one entity from the in-house gold standard in every unit and a turker had to mark at least one entity to submit) and including empty units (ie, units that have no entities from the in-house gold standard). To mirror the original task given to the traditional annotators and to keep the annotated sample representative of the full CTA corpus, we kept the empty units at 30% of the crowdsourced units.

During the pilot annotations, we had difficulty with a large numbers of untrusted turkers and judgments coming from Asia so we restricted the project to turkers from Australia, Canada, the United Kingdom, and the United States. We also requested 5 judgments per unit (from 5 different turkers) in order to allow flexibility with voting measures and methods. In addition to the training mode, a qualification quiz was presented to each turker the first time they signed up for our tasks. They had to read and understand the instructions, and answer a short quiz (3 multiple choice questions) in order to gain access to the job. The quiz blocked “robot scripts” from participating in our tasks.

**Figure 2:**
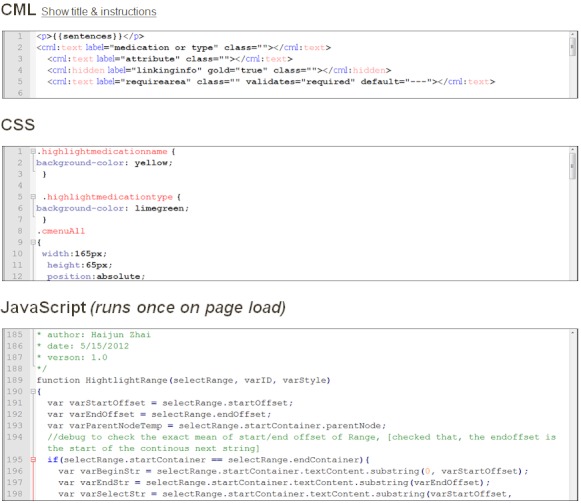
Snippets of CML, CSS and JavaScript.

**Figure 3:**
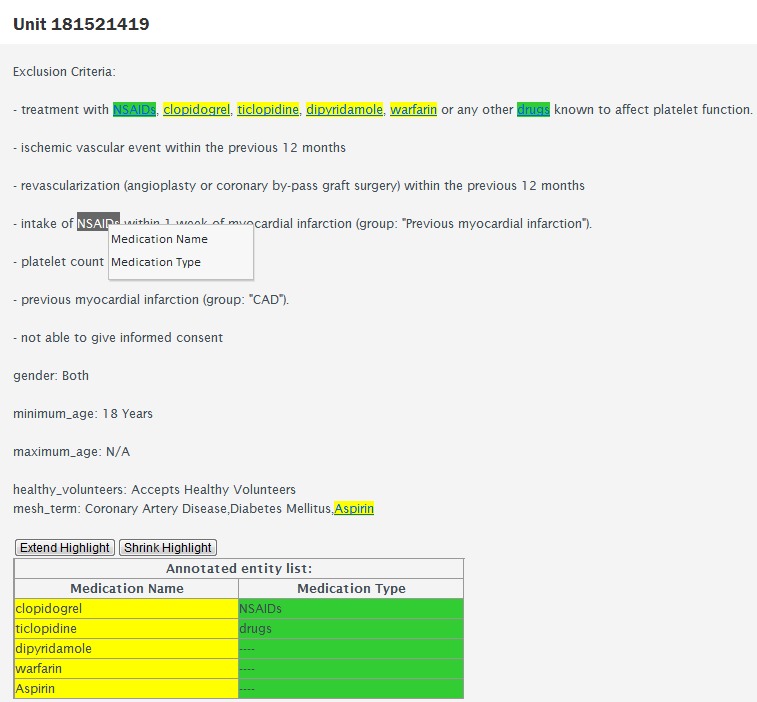
Medication named entity recognition task interface.

Figure 2 shows snippets of the corresponding CML code, common style sheet (CSS) code, and custom JavaScript. As seen in Figure 2, CF provides the interface for users to edit CML, CSS, and JavaScript in corresponding text areas named CML, CSS, and JavaScript, respectively. The complete GUI code can be found in the Multimedia Appendix 1. This interface was primarily used for creating our annotation program. The main restriction for the custom JavaScript code is that it runs only once, when CF randomly selects a unit and presents it to a turker to perform a judgment. In order to deal with this restriction, we created the program based on event-driven programming, in which each user’s annotation operation was captured and processed by a designated function. This dynamic JavaScript program worked for each unit submitted for judgment. For the medication and medication type named-entity annotation tasks, our program displayed the unit, allowing for an offset (a single word or group of words) to be selected with a left-click and drag of the mouse or a double-click and a subsequent right click event, in which the turker would select the class associated with the highlighted text. The program kept a sorted record of the offsets, classified for both entity classes, and submitted these offsets as named entities when the turker clicked “submit”. The program also handled discontinuous entities, as described below. The performance evaluation described in the experiments section involves comparing the offsets submitted between the turker’s judgments and the gold standard. Figure 3 shows the interface of the medication and medication type named-entity annotation task. In this interface, 4 of the major functions were provided, which were “word selection”, “annotation selection”, “annotation information display”, and “discontinuous highlighting”.

The “word selection” function supported double-clicking to select a single word, automatic word-extending and invalid character-shrinking to improve the accuracy and efficiency of the turkers’ operations. Two buttons (“extend highlight” and “shrink highlight”) were provided to extend and shrink the highlighted (annotated) area on the right hand side by one character at a time. After selecting one word or more, a menu with two options (“Medication Name” and “Medication Type”) popped up for the turkers to select the target annotation type. After the turkers clicked the option, the selected word(s) was highlighted by a corresponding color (eg, green was for “Medication Type,” as shown in Figure 3) and all the current annotation information was displayed in the table named “Annotated Entity List”. If the turkers needed to remove annotations already highlighted with a label or if they wanted to change the label of the highlighted word, they had to left-click on the highlighted word and click “OK” to confirm their choice to remove the annotation from the table at the bottom of the page. It should be noted that entities comprised of discontinuous tokens could be highlighted as a single entity by concurrently pressing down CTRL.

The interface for the correction task (correcting previously annotated entities) was similar to the annotation task with the only difference being that some words were pre-annotated (highlighted). The offsets associated with these highlighted words were prefilled into the unit judgment table.

**Figure 4:**
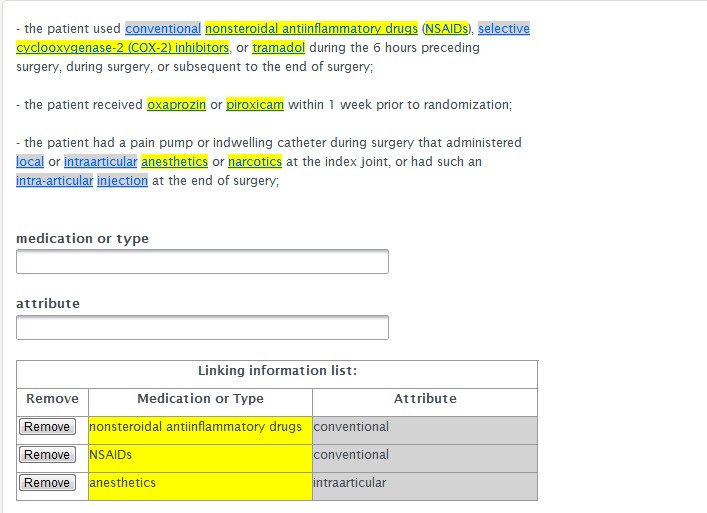
Linking task interface.

Figure 4 shows the interface for the linking task. In this interface, all medications (medication names and medication types) and attributes were highlighted with their respective colors. Medications were highlighted with yellow and attributes were highlighted with light gray. Turkers had to left click on the medications and attributes in corresponding pairs. The selected medications and attributes were displayed in the corresponding textbox to link them together and the linked pairs were shown in the table named “Linking Information List”. If the turkers wanted to remove an entity-attribute pair, they could click the "Remove" button to the left of the pair in the “Linking Information List.” In all of these tasks, the results were internally represented by offsets instead of the original text, which was necessary to address the problem of words occurring more than once in the same annotation unit. The GUI worked in Firefox and Chrome browsers. The JavaScript checked the browser type when a turker signed up for our tasks and, if the turker did not use one of the two browsers, it would instruct the turker to download and install a correct browser.

### Experiments

After the initial, smaller pilot experiments, we selected a larger number of units for the complete named-entity recognition task. In an earlier unpublished project to develop a machine learning-based medication entity-extraction pipeline, we determined that 1042 CTAs were necessary for the training set to achieve higher than 0.80 F-measures (0.86 for medication name and 0.82 for medication type, using Conditional Random Fields algorithm for information extraction). We used this empirically determined corpus size of 1042 CTAs, corresponding to 3400 units as mentioned in previous section for both the medical name-entity recognition and entity-linking jobs. Several samples annotated by turkers and their corresponding gold standard are presented in the Multimedia Appendix 2.

Based on the pilot medication named-entity annotation experiments, the correction experiment was performed by taking a smaller data set with 200 units and its corresponding 1000 judgments (5 judgments for each unit) and submitting the unique judgments to another crowdsourcing job. The previous experiment had 735 unique judgments (out of 1000). If a particular unit had 3 unique judgments and two additional duplicate judgments, we resubmitted only the 3 unique judgments for the correction job. A judgment was defined by the response of a turker to a unit, covering all of the entities annotated for that unit. In this example, the original job had 5 judgments for the unit and the correction job had 15 judgments for that same unit (3 unique judgments submitted for 5 correction judgments each). For each correction judgment, a turker had the opportunity to remove annotations, add additional annotations, or provide no change to that unit.

### Measurements

In this paper, standard named-entity recognition and classification measurements were adopted to evaluate the performance of the experiments, including Precision (P), Recall (R), and F-measure (F), which are defined in the Multimedia Appendix 3.

### Voting Methods

One of the aims of this study was to evaluate different methods of voting on judgments from crowdsourced outputs. Because these are named-entity and linking tasks, the calculation is on the entity and linking level. We experimented with 3 voting methods for the medication name and medication-type entity recognition and the medication attribute linking tasks.

We investigated 3 voting methods: simple voting (simple), trusted score weighted voting (trust), and turker experience weighted voting (experience). All voting was performed at the entity and linking level (micro average), regardless of the number of entities and linkages in a given task unit. Equations (1), (2), and (3) shown in Multimedia Appendix 4 describe the formulas we used for the 3 voting methods. Let *e* be the number of votes for a particular named entity and let *J* be the number of judgments (number of turkers who submitted responses) in this unit. Let *t*_*i*_ be the trust score of turker *i* who annotated the entity. Let *u*_*i*_ be the total number of judgments user *i* performed and let *m* be the maximum number of judgments the most prolific turker performed. For simple voting presented in Equation 1, if there were 2 or more annotations (out of 5 judgments/responses) for a particular entity, it was selected for the adjudicated judgment.

**Figure 5:**
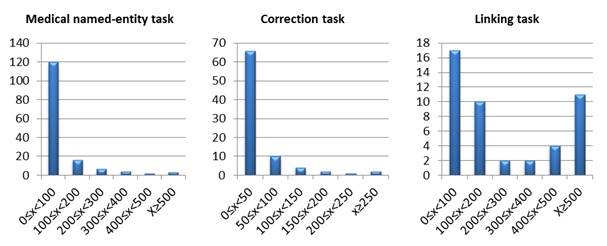
The distributions of turkers’ experience for medical named-entity task, correction task and linking task (X axis denotes number of jobs, Y axis indicates number of turkers).

Equation 2 gives the trusted score voting, which weighs a particular turker’s entity vote with their trust score (a trust score of 75% provided a 0.75 vote per entity and the max trust score of 100% provided a single simple vote). As presented in Equation 3, turker experience voting weighted each entity vote by the experience score of the turker. The experience score is the number of judgments performed by a turker relative to the maximum number of judgments the most prolific turker performed in that experiment. For example, in one job, a turker submitted 163 judgments, which was the most of any turker in that job. That turker’s weight for all of his entity votes became 1 and the experience score for all other turkers became u/163. Note that the intention of division in 3 equations was to normalize the scores to the range of 0 to 1. As presented in Figure 5, there was high variance in the accomplished number of jobs between turkers. The point of the logarithm in Equation 3 was to scale the difference.

**Figure 6:**
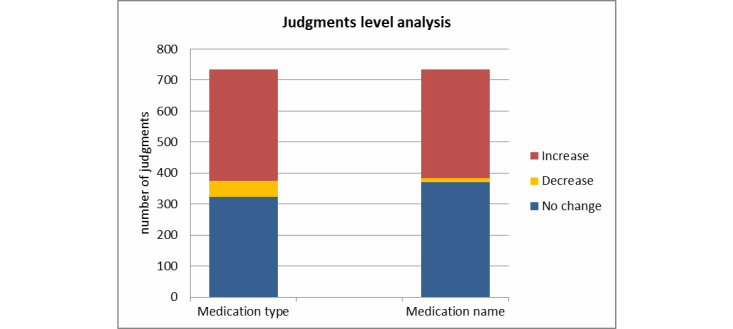
Improvement chart for correction task.

The F-measures were calculated using the 3 voting methods on the original judgments (with each unit having 5 judgments) as correction baselines presented in Table 5. These were then compared to the subsequent correction results computed by 3 voting methods of all correction judgments presented in Table 6. In order to further show the impact of correction, another measure, which we described as a response-level entity vote, is presented in Figure 6. We counted whether the F-measure of the correction judgments improved upon the F-measure of the original judgment.

### Statistical Significance Test of Turker Performance

In order to analyze the differences between the corpus created by the turkers and the corpus created by in-house expert annotators, a statistical assessment method (named “pooling chi-square test”) was proposed to calculate the *P* values. In this method, the voting results from turkers were pooled together with the corpus that was annotated by experts. These pooled results were then tested against the original voting results. The hypothesis was that the turkers with sufficient training and aggregating multiple results could perform as well as experts. If this hypothesis was true, then pooling the results was not expected to change the original CF voting results. Specifically, the hypothesis H_0_ was that the experts’ output did not change the quality of the turkers’ annotations (reflected by the number of unique entities annotated correctly and incorrectly). If the *P* value was less than the designated threshold (0.05), it meant that the experts’ output significantly affected the quality of the turkers’ results. In other words, the turkers did not perform as well as experts. If the *P* value was higher than the predetermined threshold, then we could not reject the hypothesis. Therefore, we could infer that there was no evidence for statistically significant differences between the turkers’ and experts’ annotations.

## Results

### Information on Turkers

**Figure 7:**
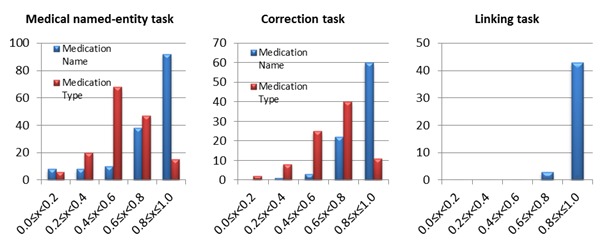
The distribution of turkers’ F-measure for medical named-entity task, correction task and linking task (X axis denotes F-measure, Y axis indicates number of turkers).

Table 2 shows information on turkers participating in our 3 tasks. We had 156 turkers, 86 turkers, and 46 turkers to complete medical named-entities task, correction task and linking task, respectively. Figure 5 presents the distribution of turkers by the number of performed jobs for the 3 tasks. We found that the top 5 most prolific turkers completed 39.9% (6778/17,000) medical named-entities jobs, 44.0% (1616/3675) correction jobs, and 45.4% (7716/17,000) linking jobs. Figure 7 shows the distribution of F-measure of turkers for the 3 tasks. We can see that F-measures of greater than 0.6 were achieved by over 83% turkers for the medical named-entities task, over 88% of turkers for the correction task and 100% for the linking task. Table 3 presents the cost and completion time of the 3 tasks. The payment of 3.84 cents per judgment included 3 cents paying for turkers and 0.84 cents charged by CF. Table 3 also presents the time required for the in-house annotators to complete the same tasks. Additionally, the time to receive results from in-house annotation is around 5 times longer than crowdsourcing due to the parallel nature of the crowdsourcing task and the traditional work hours (eg, Monday to Friday, 9am-5pm). The 133 hours represented by the total in-house annotation were the total work hours. The total elapsed time was 10 days (8 work days plus 2 weekend days).

### Results of Medical Named-Entities Annotation Task

Table 4 shows the results of the turkers’ medical named-entity annotation with the 3 voting methods that were implemented. It shows the turkers’ generated corpus’ agreement with the in-house experts’ generated gold standard at various threshold levels.

**Table 2:** Information on turkers participating in the 3 tasks.

Task name	Participating turkers	Turkers passing the test
Medical named-entities	1144	156
Correction	678	86
Linking	644	46

**Table 3:** Cost and time of the 3 tasks.

		Crowdsourcing			In-house	
Task name	Total units	Total judgments	Total cost (per judgment)	Total time (per judgment)	Total judgments	Total time (per judgment)
Medical named-entities	3400	17,000	$652.85 (3.84 cents)	57 hours (12.07 seconds)	6800	128 hours (67.76seconds)
Correction	735	3675	$141.13 (3.84 cents)	38 hours (37.22 seconds)	N/A	N/A
Linking	3400	17,000	$652.85 (3.84 cents)	27 hours (5.72 seconds)	6800	44 hours (23.29 seconds)

**Table 4:** Results of medical named entity annotation (the pre-determined threshold and its corresponding P^a^, R^b^, and F^c^ for each column are italicized).

Simple						Trust				Experience			
Th^d^			P	R	F	Th	P	R	F	Th	P	R	F
**Medication name**													
		0.20	0.694	0.931	0.796	0.18	0.835	0.887	0.860	0.18	0.807	0.895	0.849
		*0.40*	*0.864*	*0.879*	*0.871*	*0.24*	*0.864*	*0.879*	*0.871*	*0.24*	*0.869*	*0.874*	*0.872*
		0.60	0.920	0.815	0.865	0.30	0.869	0.874	0.871	0.30	0.885	0.854	0.870
		0.80	0.955	0.696	0.805	0.36	0.916	0.819	0.865	0.36	0.910	0.820	0.863
**Medication type**													
	0.20		0.431	0.879	0.579	0.18	0.632	0.781	0.699	0.18	0.583	0.800	0.675
	*0.40*		*0.698*	*0.763*	*0.729*	*0.24*	*0.698*	*0.763*	*0.729*	*0.24*	*0.711*	*0.751*	*0.731*
	0.60		0.831	0.598	0.696	0.30	0.709	0.745	0.727	0.30	0.756	0.703	0.729
	0.80		0.911	0.396	0.552	0.36	0.819	0.608	0.698	0.36	0.816	0.614	0.700

^a^precision

^b^recall

^c^F-measure

^d^threshold

### Results of Correction Task

The results of the correction task, its corresponding correction baseline, and the results of combined judgments are presented in Tables 5 and 6, respectively. In the correction task, the turkers and experts agreement F-measure of medication name and medication type achieved 0.900 and 0.760 by simple vote, respectively. With comparison to the F-measure of its corresponding correction baseline, relative improvements of 2.62% (medication name Baseline F-measure = 0.877, After_Correction_F-measure = 0.900; computed by (After_Correction_F-measure - Baseline_F-measure)/ Baseline_F-measure * 100) and 10.79% (n/N; medication type name Baseline_F-measure = 0.686, After_Correction_F-measure = 0.760; computed by (After_Correction_F-measure - Baseline_F-measure)/ Baseline_F-measure * 100) were gained (Tables 4 and 5).

**Table 5:** Results of correction task with 200 units and 1000 judgments (the pre-determined threshold and its corresponding P^a^, R^b^, and F^c^ for each column are italicized).

Simple					Trust				Experience			
Th^d^		P	R	F	Th	P	R	F	Th	P	R	F
**Medication name**												
	0.20	0.796	0.938	0.861	0.18	0.825	0.927	0.873	0.18	0.845	0.921	0.881
	*0.40*	*0.896*	*0.904*	*0.900*	*0.24*	*0.861*	*0.916*	*0.888*	*0.24*	*0.876*	*0.908*	*0.892*
	0.60	0.950	0.812	0.875	0.30	0.898	0.906	0.902	0.30	0.900	0.891	0.896
	0.80	0.972	0.732	0.835	0.36	0.908	0.887	0.897	0.36	0.933	0.867	0.899
**Medication type**												
	0.20	0.610	0.916	0.733	0.18	0.655	0.892	0.755	0.18	0.662	0.872	0.752
	*0.40*	*0.732*	*0.790*	*0.760*	*0.24*	*0.691*	*0.843*	*0.759*	*0.24*	*0.703*	*0.817*	*0.756*
	0.60	0.851	0.541	0.661	0.30	0.736	0.792	0.763	0.30	0.756	0.773	0.764
	0.80	0.945	0.382	0.544	0.36	0.776	0.744	0.760	0.36	0.783	0.684	0.730

^a^precision

^b^recall

^c^F-measure

^d^threshold

**Table 6:** Baseline Results of medical named entity annotation corresponding to the correction task (the pre-determined threshold and its corresponding P^a^, R^b^, and F^c^ for each column are italicized).

Simple					Trust				Experience			
Th^d^		P	R	F	Th	P	R	F	Th	P	R	F
**Medication name**												
	0.20	0.712	0.934	0.808	0.18	0.839	0.891	0.864	0.18	0.774	0.908	0.835
	*0.40*	*0.868*	*0.887*	*0.877*	*0.24*	*0.868*	*0.887*	*0.877*	*0.24*	*0.872*	*0.887*	*0.879*
	0.60	0.909	0.788	0.844	0.30	0.870	0.887	0.878	0.30	0.881	0.876	0.879
	0.80	0.956	0.655	0.778	0.36	0.899	0.803	0.848	0.36	0.894	0.809	0.849
**Medication type**												
	0.20	0.473	0.879	0.615	0.18	0.627	0.724	0.672	0.18	0.594	0.779	0.674
	*0.40*	*0.669*	*0.704*	*0.686*	*0.24*	*0.669*	*0.704*	*0.686*	*0.24*	*0.668*	*0.698*	*0.683*
	0.60	0.737	0.519	0.609	0.30	0.670	0.700	0.685	0.30	0.681	0.664	0.673
	0.80	0.890	0.358	0.510	0.36	0.726	0.550	0.626	0.36	0.717	0.558	0.628

^a^precision

^b^recall

^c^F-measure

^d^threshold

Furthermore, we analyzed the practical significance of these improvements by calculating the F-measure of medication name and medication type for each unique judgment (the total number of unique judgments was 735) and its corresponding 5 correction judgments. Based on empirical evidence acquired in previous experiments, the F-measure was computed based on a simple vote with the threshold of 0.4. The results are shown in Figure 6. Improvement was seen for 50.5% (370/735) and 44.1% (324/735) of the judgments for medication name and medication type after the turkers’ correction, respectively. In contrast, 1.9% (14/735) and 6.9% (51/735) judgments became worse.

### Result of Linking Task

Table 7 shows the results of the linking experiment. Non-expert annotators (turkers) did an excellent job, in which the F-measure achieved 0.962. Meanwhile, as previous results indicated, the simple method could obtain very good results in case of strict quality control.

**Table 7:** Results of linking task (the pre-determined threshold and its corresponding P^a^, R^b^, and F^c^ for each column italicized).

Simple				Trust				Experience			
Th^d^	P	R	F	Th	P	R	F	Th	P	R	F
0.20	0.845	0.984	0.910	0.18	0.927	0.982	0.954	0.18	0.927	0.979	0.952
*0.40*	*0.949*	*0.975*	*0.962*	*0.24*	*0.949*	*0.975*	*0.962*	*0.24*	*0.950*	*0.974*	*0.962*
0.60	0.981	0.959	0.970	0.30	0.949	0.975	0.962	0.30	0.955	0.973	0.964
0.80	0.990	0.925	0.956	0.36	0.975	0.967	0.971	0.36	0.977	0.965	0.971

^a^precision

^b^recall

^c^F-measure

^d^threshold

### Results of Statistical Significance Analysis

For all the results above, Chi-square statistical significance tests were conducted between the corpora created by Crowdflower’s and the gold-standard generated by the in-house annotators. The *P* values (at *P*<.001) showed no statistically significant difference between the best CrowdFlower generated corpora and corresponding in-house generated gold-standard sets.

## Discussion

### Overview

To our knowledge, the medical named-entity annotation task described in this work is the largest scale crowdsourcing experiment in the clinical NLP research field. The results demonstrated that crowdsourcing is a feasible solution for creating a gold standard for medical named-entities. Many works were described in the introduction section, but only one performed a similar medical named entity crowdsourced annotation and is directly comparable to our current study. All other works focused on different corpora and entity types and cannot be compared directly with these works. We improved upon the previously reported results on medical named entity annotation task [21,22] with more than 27.9% of the F-measure (F-measure_Current_Study = 0.87 vs F-measure_Earlier_Work = 0.68 for agreement between the crowdsourced and traditionally developed corpora; computed by (F-measure_Current_Study vs F-measure_Earlier_Work)/ F-measure_Earlier_Work * 100) for named-entity annotation. This experiment also showed that the crowdsourcing performance for medication name annotations is much better than those of medication type. This is a similar finding to the in-house results with trained, expert annotators. We attribute this phenomenon to the clarity of the task for medication name annotation. In other words, the definition and the gold standard answers of medication names are easier to understand and to capture than those of medication type. In the future, we plan to use a more easily interpretable definition of medication types to improve performance. We also plan to use crowdsourcing to annotate attributes, such as date, dosage, as listed in Table 1.

Based on our experiments, we found that it was easy to find a large number of turkers by crowdsourcing. Around 10% (156/1144, 86/678, 46/644 for medication name entity, linking and correction tasks, respectively) of the turkers passed our quality control test (see Table 2). Among those turkers, around 10% (14/156, 11/86, 7/46) of them contributed over 50% (10,521/17,000, 10,900/17,000, 1907/3675) of the jobs.

As shown in Table 4, the non-expert annotators performed at a very high quality and the results indicated that the simple method could obtain very good results, provided the quality control is strict. In our previous work [29], we reported inter-annotator agreement (IAA) F-measures for medication names and medication types, 94.2% and 88.2% respectively. Additionally, what could have conceivably been weeks’ worth of in-house annotation work was achieved in less than a day of crowdsourcing effort.

Our previous study conducted experiments by implementing a rule-based linking system [31]. The result (around 0.72 F-measure) showed that manual annotation is definitely needed to develop an effective training set for a machine learning-based linking system. The presented linking experiment is the first work known to us that attempted to link medications to their corresponding attributes with crowdsourcing. The results indicated that linking is not a difficult task and the data created can be sufficiently applied to real applications. Based upon this experiment, we plan to create a larger scale data set using crowdsourcing and to apply it to clinical NLP tasks. We will further evaluate the performance of linking by implementing our linking strategy for other clinical named entities. The results of the linking task are excellent, with a near 100% (N=3400) agreement between crowd and traditionally developed corpora.

As shown in Table 3, the linking task took much less time than the other two tasks, most likely because the linking task is much easier than the other two tasks. The time per judgment for medical named entity annotation task is much less than that of the correction task (12.07 vs 37.22 seconds respectively). The reason is that the medical named entity annotation task has more participating turkers (156 vs 86). We can conclude that the difficulty of tasks and the number of participating turkers strongly affect the completion time of the tasks. In contrast to traditional annotation, crowdsourcing achieved 55.5% time (71/128 hours) and 75.0% cost ($1958/$2611) savings for medical named entity annotation. For the linking task, 38.6% time (17/44 hours) and 27.2% cost ($244/$897) savings were seen when using crowdsourcing.

To our knowledge, we were the first to conduct clinical NLP correction experiments. The results of that experiment are quite encouraging. Our correction F-measure was 0.90 (medication names) and even the worst final F-measure improved by more than 10% after the corresponding voting (medication types). We believe that this experiment showed another feasible and efficient way to improve the output of crowdsourcing. We designed an efficient strategy to perform correction. Future work will focus on determining the number of iterative cycles to achieve the best results.

As was mentioned in the previous sections, creating a smaller batch of gold standard data (in-house with expert annotators) is a critically important step for crowdsourcing quality control. This in-house gold standard can be used later to: (1) train turkers, (2) perform quality control, and (3) determine thresholds to aggregate judgments. In this study, we also modified the gold standard management interfaces of CF to perform turker training and quality control by setting gold standard answers. There is room for further research in different methods to train turkers and to experiment with quality control thresholds.

Finally, 3 different voting methods were investigated to aggregate judgments. The results showed that it is quite possible to acquire a high-quality annotated corpus by implementing simple voting under the condition of strict quality control. In pilots, we experimented with different voting thresholds. Lower thresholds resulted in lower agreement of the turker-annotated corpora with the gold standard corpora. Higher thresholds prevented the successful completion of the task by eliminating too many turkers. The thresholds used in the paper (eg, 2 judgments out of 5 or 0.4) were set empirically based on our pilot experiments and earlier related work [32,33]. For the judgment-based voting (eg, trust-based and experience-based voting) more complicated voting methods could be implemented and compared.

A potential limitation of this study was that, the proportion of empty units in our experimental corpus was less (30%) than that in the general population of CTA documents (42%). On the other hand, our pilot experiments show that the proportion of empty units did not influence the performance of the turkers. A second potential limitation was that we included only 3 voting methods among the tested voting schemas. We plan to address this limitation in our future works.

### Conclusions

In this study, we evaluated the feasibility of crowdsourcing for creating gold standard data for clinical NLP tasks. Although direct comparison with all related work in the literature was not possible because of corpora and entity type differences, by implementing strict quality control for turker selection and by continuously monitoring the turkers’ performance, we improved upon the directly comparable results in the literature with more than 27.9% for the named-entity annotation task. 3 major experiments were conducted: (1) named-entity annotation, (2) entity linking, and (3) annotation correction. In addition, 3 voting methods were studied. To our knowledge we were the first to investigate the feasibility of crowdsourcing for clinical named-entity annotation on a large-scale corpus. Similarly, we are not aware of a competing work in the clinical NLP domain that proposed to use crowdsourcing to create an entity-linking gold standard for information extraction, on our experiments’ scale. Furthermore, we proposed a successful correction strategy that applied crowdsourcing to crowdsourcing results to improve the quality of the annotated corpus. We found that a high-quality, clinical NLP gold standard data could be obtained by a simple voting method, if a strict quality control is implemented.

Based upon the results of our experiments, we conclude that crowdsourcing is a feasible, inexpensive, fast, and practical approach to annotate clinical text (when protected health information is not included) on large scale for medical named-entities. We believe that well-designed user interfaces for entity annotation and linking were critical to the success of this work. As a further contribution to the Web 2.0-based crowdsourcing field, we publicly released the JavaScript and CML infrastructure code that is necessary to utilize CrowdFlower’s quality control and crowdsourcing interfaces for named entity annotations [34].

## Multimedia Appendix 1

GUI source code.

## Multimedia Appendix 2

Samples annotated by turkers and their corresponding gold standard.

## Multimedia Appendix 3

Definitions of precision, recall, and F-measure.

## Multimedia Appendix 4

Voting method equations.

## Abbreviations

AMT: Amazon Mechanical Turk
CF: CrowdFlower
CML: CrowdFlower Markup Language
CSS: common style sheet
CTA: clinical trial announcement
EMU: Extractor of Mutations
GUI: graphical user interface
IAA: inter-annotator agreement
MEDLINE: Medical Literature Analysis and Retrieval System Online
NCBI: National Center for Biotechnology Information
NLP: natural language processing
