# Supplementary material for: Web 2.0-Based Crowdsourcing for High-Quality Gold Standard Development in Clinical Natural Language Processing
Source: J Med Internet Res. 2013 Apr 2;15(4):e73. doi: 10.2196/jmir.2426 (PMC3636329; doi:10.2196/jmir.2426)
Supplement: Supplementary file 1 [file jmir_v15i4e73_app1.pdf]

GUI Source Code **(original source code can be found on https://code.google.com/p/soltilab/)**

1. GUI code for named entity annotation

```
<!--

###############################

#Main Developer: Haijun Zhai, PhD


# Contributor: Todd Lingren, MA

# Supervised by: Imre Solti, MD, PhD, MA

# Contact: Haijun.Zhai@cchmc.org

# Open Source License: Apache 2.0

#

# Date: 08/01/2012

# Created: 08/01/2012

# Modified: 08/01/2012 (Imre Solti)

#

###############################

# License

########################

Copyright 2012 Imre Solti

Licensed under the Apache License, Version 2.0 (the "License");

you may not use this file except in compliance with the License.

You may obtain a copy of the License at

http://www.apache.org/licenses/LICENSE-2.0

Unless required by applicable law or agreed to in writing, software

distributed under the License is distributed on an "AS IS" BASIS,

WITHOUT WARRANTIES OR CONDITIONS OF ANY KIND, either express or implied.

See the License for the specific language governing permissions and
```

limitations under the License.

-->

```
<html><style type="text/css" media="screen">
#selectioncontent {
    border: solid black 1px;
    padding: 0.125em;
    background-color: white;
    overflow: auto;
}


.highlightmedicationname {
background-color: yellow;
 }

 .highlightmedicationtype {
background-color: limegreen;
 }
.cmenuAll
{
 width:165px;
  height:65px;
  position:absolute;
}
.cmenu
{
  margin:0px 5 5 0px;
```

```
  z-index:10;
  width:160px;
  height:60px;
  background-color:#F7F7F7;
  border:1px solid #BFBFBF;
  font-family:"Calibri";
  position:absolute;

}
.cmenuBack
{
  margin:2px 0 0 2px;
  width:160px;
  height:60px;
  background-color:#CCCCCC;
  border:2px solid #E3E3E3;
  position:absolute;
}
.liAble
{
  font-family:"Calibri";
  color:#252C2B;
  margin-left:3px;
  margin-top:5px;
  list-style-type:none;
  cursor:default;
}
.liMouseOver
```

```
{
  margin-left:3px;
  margin-top:5px;
  background-color:#D1E0FE;
  border:1px solid #6699FF;
  list-style-type:none;
  cursor:default;
}
.divTableAnnotationRes
{
	float:top;
	width:500px;
	border-left:#999999 1px solid;
	border-top:#999999 1px solid;
	display:table;
}
.divTableAnnotationRes_Title
{
	float:left;
	width:498px;
	list-style:none outside;
	border-bottom:#999999 1px solid;
	border-right:#999999 1px solid;
	border-left:#999999 1px solid;
	text-align:center;
	font:border;
	list-style:none;
	font-weight:900;
```

```
}
.divTableAnnotationRes_Head_C1
{
	float:left;
	width:49px;
	border-left:#999999 1px solid;
	border-right:#999999 1px solid;
	border-bottom:#999999 1px solid;
	font:border;
	text-align:center;
	list-style:none;
	font-weight:700;
}
.divTableAnnotationRes_Head_C2
{
	float:left;
	width:248px;
	border-left:#999999 1px solid;
	border-right:#999999 1px solid;
	border-bottom:#999999 1px solid;
	font:border;
	text-align:center;
	list-style:none;
	font-weight:700;
}
.divTableAnnotationRes_Head_C3
{
	float:left;
```

```
	width:249px;
	border-right:#999999 1px solid;
	border-bottom:#999999 1px solid;
	font:border;
	text-align:center;
	list-style:none;
	font-weight:700;
}
.divTableAnnotationRes_Col1
{
	float:left;
	width:49px;
	border-left:#999999 1px solid;
	border-right:#999999 1px solid;
	border-bottom:#999999 1px solid;
	font:border;
	text-align:left;
	list-style:none;

}
.divTableAnnotationRes_Col2
{
	float:left;
	width:248px;
	border-left:#999999 1px solid;
	border-right:#999999 1px solid;
	border-bottom:#999999 1px solid;
	font:border;
```

```
		text-align:left;
		list-style:none;
		background-color:yellow;
}
.divTableAnnotationRes_Col3
{
		float:left;
		float:left;
		width:249px;
		border-right:#999999 1px solid;
		border-bottom:#999999 1px solid;
		font:border;
		text-align:left;
		list-style:none;
		background-color:limegreen;
}

.divSelectionTool
{
		float:top;
		width:240px;
		display:table;
}
</style>
<body>
<p><b>Steps</b> <span style="color:blue" onclick="CCHMCShowDetail('CCHMC2')">More</span></p>
<p id="CCHMC2" style="display:none;">
```

Step 1: Select the word or group of words to be labeled. Double click on a word or click the left mouse button and drag the mouse to select/highlight the word. You can hold down the left mouse button and move the mouse to select/highlight more than one word.

Step 2: Click the right mouse button on the word(s) selected and a menu will pop up.

Step 3: Select the label option from the pop up menu. If this operation is successful, the word(s) will be highlighted by a corresponding color (medication_name is yellow and medication type is green). The word will also automatically be added to a table at the bottom of the page.

Removing Labels: If you wish to remove a word you already highlighted with a label or if you want to change that label of the highlighted word, then you need to left click on the highlighted word and click 搊 k?to confirm your choice. It will be removed from the table at the bottom of the page. If you do not wish to remove, click 挿 ancel?

Modifying Labels: If you wish to extend the words or letters in a selected word, and you have already selected the label for the word(s), please remove it and start over with Step 1.

Shrinking/Extending Words: If you have selected the word or words (Step 1) and have not selected the label (Step 2 and 3), then click 揺 xtend word?to add a character to the right of the selected word. Click 揝 hrink word?to unselect one character from the right side.

Some medications and medication types will not be continuous words. For example:

   <i>hormonal and barrier method of birth control</i>

   "hormonal" "method of birth control" is a medication type.

   Label "hormonal" as a medication type. Then highlight "method of birth control" and right click on the highlighted words. Cntrl-left click on the label and it will add this group of words to the medication type you just added ("hormonal").

</p>

```
<div class="cml jsawesome"
id="u0100025156115077098153154121160060073211009108245072045038141154136039065175144
095183245160093121"><p>          7. Signed and dated written informed consent by date of Visit 1a in
accordance with GCP<br>            and local legislation<br><br>       Exclusion Criteria:<br><br>         1.
Myocardial infarction, stroke or TIA within 6 months prior to informed consent<br><br>         2.
Impaired hepatic function<br><br>         3. Known hypersensitivity or allergy to the investigational
product or its excipients or<br>            metformin or placebo<br><br></p><div class="hidden
cml_field"><input
name="u0100025156115077098153154121160060073211009108245072045038141154136039065175
144095183245160093121[medicationname]" type="display" value="No data available"
class="medicationname" /></div>

  <div class="hidden cml_field"><input
name="u0100025156115077098153154121160060073211009108245072045038141154136039065175
144095183245160093121[medicationtype]" type="display" value="[350-373,];[421-428,];

" class="medicationtype" /></div>

       <div class="text cml_field"><label class="legend">medicationnamemedicationtype</label>

<div class="cml_row"><input
name="u0100025156115077098153154121160060073211009108245072045038141154136039065175
144095183245160093121[medicationnamemedicationtype]" type="text" value=""
class="medicationnamemedicationtype validates-required" /></div>

</div>



</div>

  </body>

</html>

<script type="text/javascript">

//globe variable for counting total entities highlighted by now (used as id for each entity)

var varStaticHighlightCount = 0;

//var for map the relations between highlight_id and resultmap_id, entry format  [highlight_id,
resultmap_id]; note that resultmap_id may be repeated

var varStaticMapHighlightToResult = new Array();

//control tag options menu

//active DIV

var varStaticDIVID;
```

```
String.prototype.rtrim = function() {
	return this.replace(/[\s.,;!\?]+$/,"");
}
//trim space in the left
String.prototype.ltrim = function() {
	return this.replace(/^\s+/,"");
}
//test if or not an char is an alphabet and digit.
function IsAlphabetORNum(sChar)
{
	var varAlphabetAndNum = /[0-9a-zA-Z]/;
	if(varAlphabetAndNum.test(sChar))
		return true;
	return false;
}

//test if or not the char is a valid begin of terms
function IsValidBegin(sChar)
{
	return IsAlphabetORNum(sChar);
}
//test if or not the char is a valid end of terms
function IsValidEnd(sChar)
{
	return IsAlphabetORNum(sChar);
}
```

```
//return the index of the first occurance of the non-AlphabetORNum backward.
function LastIndexofNonAlNum(sStr)
{
	//return sStr.search(/\s[^\s]*$/);
	return sStr.search(/[0-9a-zA-Z]+$/);
}

//return the index of the first occurance of the AlphabetORNum backward.
function LastIndexofAlNum(sStr)
{
	return sStr.search(/[0-9a-zA-Z][^0-9a-zA-Z]*$/);
}

//return the index of the first occurance of the non-AlphabetORNum afterward.
function FirstIndexofNonAlNum(sStr)
{
	return sStr.search(/[^0-9a-zA-Z]/);
}

//return the index of the first occurance of the AlphabetORNum afterward.
function FirstIndexofAlNum(sStr)
{
	return sStr.search(/[0-9a-zA-Z]/);
}

//global variable save if or not it is clicked on tag options
var varStaticOptionVisible = false;
//global variable for store the selected range
```

```
var varStaticCurrentSelectRange;



//API for implementing highlight


/*function: calculate the offset in the original text for the highlights.
* input: selectRange[Range: give the current text selection]
* output: varOffset[int: the offset in the originaltext for the hightlights]
* author: Haijun Zhai
* date: 5/15/2012
* verson: 1.0
*/
function CalculateStartOffset(selectRange)
{
	var varOffset = 0;

	var varPreviousSibling = selectRange.startContainer.previousSibling;
	while(varPreviousSibling){
		if(varPreviousSibling.tagName == "BR") varOffset ++;
		else varOffset += varPreviousSibling.textContent.length;
		varPreviousSibling = varPreviousSibling.previousSibling;
	}
	return (varOffset + selectRange.startOffset);
}
//API for implementing highlight


/*function: calculate the offset in the original text for the hightlights.
* input: selectRange[Range: give the current text selection]
```

```
* output: varOffset[int: the offset in the originaltext for the hightlights]

* author: Haijun Zhai

* date: 5/15/2012

* verson: 1.0

*/

function CalculateEndOffset(selectRange)

{

	var varOffset = 0;


	var varPreviousSibling = selectRange.endContainer.previousSibling;

	while(varPreviousSibling){

		if(varPreviousSibling.tagName == "BR") varOffset ++;

		else varOffset += varPreviousSibling.textContent.length;

		varPreviousSibling = varPreviousSibling.previousSibling;

	}

	return (varOffset + selectRange.endOffset);

}


/*function: remove hightlights belonging to the same anotation(entity). note that the <a> node will be concatenated with the textnode before and after into a new textnode

* input: varID[String: format medicationname/medicationtype_divid_countid", note that this id will be used as name to indentify all the hightlight belonging to one notation]

* input: varStyle[: using for seting node display style]

* author: Haijun Zhai

* date: 5/15/2012

* verson: 1.0

*/

function RemoveHiglight(varID)
```

```
{
	var varConfirmRes = confirm("Are you sure to remove this anotation?");
	if (varConfirmRes == true){
		var varIdItems = varID.split("_");
		var varElementDIV = document.getElementById(varIdItems[1]);
		var varAllNodes = varElementDIV.getElementsByTagName("a");
		var varAllResANodes = new Array();
		for(var i = 0; i < varAllNodes.length; i++){
			if(varAllNodes[i].name == varID)
				varAllResANodes.push(varAllNodes[i]);
		}

		//note that varAllResANodes.length is NOT changing dynamicly,
		for(var i = 0; i < varAllResANodes.length && varAllResANodes; i++ ){
			var varString = "";
			var varANode = varAllResANodes[i];
			var varParent = varANode.parentNode;
			if(varANode.previousSibling && varANode.previousSibling.nodeType == 3){
				varString += varANode.previousSibling.textContent;
				varANode.parentNode.removeChild(varANode.previousSibling);
			}
			varString += varANode.textContent;
			if(varANode.nextSibling && varANode.nextSibling.nodeType == 3){
				varString += varANode.nextSibling.textContent;
				varANode.parentNode.removeChild(varANode.nextSibling);
			}
			var varTextNode = document.createTextNode(varString);
			varANode.parentNode.replaceChild(varTextNode, varANode);
```

```
			}

			//varResultID [String: indicating the notations to be displayed in the correseponding
textbox, "medicationname/medicationtype"_DivID]

			//TextboxID [String: Indicating the textbox to display the notations, note that this is the
name, DivID_"[medicationame/medicationtype]"]

			varStaticMapResult.remove(varID);

			DispResInTBox(varIdItems[1], varIdItems[0]);

		}

}


/*function: create "<a>" node (including one textnode)

* input: varID[String: format divid_countid"]

* input: varStyle[String: using for seting node display style]

* input: varText[String: the selected text]

* output: varANode[Node]

* author: Haijun Zhai

* date: 5/15/2012

* verson: 1.0

*/

function CreateSpanNode(varID, varStyle, varText)

{

	//var varOnlcik = "javascript:RemoveHiglight('" + varID + "');";

	var varANode = document.createElement("a");

	varANode.setAttribute("href", "javascript:void(0);");

	varANode.setAttribute("class", varStyle);

	//varANode.setAttribute("onclick", varOnlcik);

	varANode.name = varID;

	//varANode.setAttribute("onclick", function(varID){return
function(){RemoveHiglight(varID);}}(varID));
```

```
	varANode.onclick = function(varID){return function(){RemoveHiglight(varID);}}(varID);

	var varTextNode = document.createTextNode(varText);

	varANode.appendChild(varTextNode);

	return varANode;

}



/*function: highlight selected text, by slicing one textnode into three nodes with one textnode + one highlighted <a> node(including one textnode) + one textnode

* note that if text is "", then textnode will not be created

* input: selectRange[Range: give the current text selection]

* input: varID[String: format medicationname_divid_countid", note that this id will be used as name to indentify all the hightlight belonging to one notation]

* input: varStyle[: using for seting node display style]

* output: status[boolean:valid hightlighting or not]

* author: Haijun Zhai

* date: 5/15/2012

* verson: 1.0

*/

function HightlightRange(selectRange, varID, varStyle)

{

	var varStartOffset = selectRange.startOffset;

	var varEndOffset = selectRange.endOffset;

	var varParentNodeTemp = selectRange.startContainer.parentNode;

	//debug to check the exact mean of start/end offset of Range, [checked that, the endoffset is the start of the continous next string]

	if(selectRange.startContainer == selectRange.endContainer){

		var varBeginStr = selectRange.startContainer.textContent.substring(0, varStartOffset);

		var varEndStr = selectRange.startContainer.textContent.substring(varEndOffset);
```

```
			var varSelectStr = selectRange.startContainer.textContent.substring(varStartOffset, varEndOffset);

			var varNextSibling = selectRange.startContainer.nextSibling;

			varParentNodeTemp.removeChild(selectRange.startContainer);

			if(varBeginStr.length > 0)varParentNodeTemp.insertBefore(document.createTextNode(varBeginStr), varNextSibling);

			varParentNodeTemp.insertBefore(CreateSpanNode(varID, varStyle, varSelectStr), varNextSibling);

			if(varEndStr.length > 0)varParentNodeTemp.insertBefore(document.createTextNode(varEndStr), varNextSibling);

		}else{//select more than one node

			var varStartBeginStr = selectRange.startContainer.textContent.substring(0, varStartOffset);

			var varStartEndStr = selectRange.startContainer.textContent.substring(varStartOffset);


			var varEndBeginStr = selectRange.endContainer.textContent.substring(0, varEndOffset);

			var varEndEndStr = selectRange.endContainer.textContent.substring(varEndOffset);



			var varNextSibling = selectRange.startContainer.nextSibling;

			while(varNextSibling != selectRange.endContainer){

				if(varNextSibling.tagName == "BR");	//

				else if(varNextSibling.nodeType == 3){//replace text node with "a" node

					var varStrTextNode = varNextSibling.textContent;

					varParentNodeTemp.replaceChild(CreateSpanNode(varID, varStyle, varStrTextNode), varNextSibling);

				}

				varNextSibling = varNextSibling.nextSibling;

			}

			//split the start and end node into more nodes
```

```
			//start node
			var varInsertPosNode = selectRange.startContainer.nextSibling;
			varParentNodeTemp.removeChild(selectRange.startContainer);
			if(varStartBeginStr.length > 0)varParentNodeTemp.insertBefore(document.createTextNode(varStartBeginStr), varInsertPosNode);
			varParentNodeTemp.insertBefore(CreateSpanNode(varID, varStyle, varStartEndStr), varInsertPosNode);
			//end node
			varInsertPosNode = selectRange.endContainer.nextSibling;
			varParentNodeTemp.removeChild(selectRange.endContainer);
			varParentNodeTemp.insertBefore(CreateSpanNode(varID, varStyle, varEndBeginStr), varInsertPosNode);
			if(varEndEndStr.length > 0)varParentNodeTemp.insertBefore(document.createTextNode(varEndEndStr), varInsertPosNode);

		}

		return true;
}
/*function: check if a selection is valid and shrink the "br" node at the start or(and) the tail of the selected range
* author: Haijun Zhai
* date: 6/7/2012
* verson: 1.0
*/
function IsValidSelection()
{
		var varSelection = window.getSelection();
		if(!varSelection || varSelection.rangeCount < 1) return false;
		var varSelectRange = varSelection.getRangeAt(0);
```

```
var varTestSelection = new String(varSelection.toString());

if( ((varTestSelection.rtrim().ltrim()) != "") &&

        (varSelectRange.startContainer.parentNode ==
varSelectRange.endContainer.parentNode

        || varSelectRange.startContainer.parentNode== varSelectRange.endContainer

        || varSelectRange.startContainer == varSelectRange.endContainer.parentNode)){




        //remove all the br nodes selected at the begin or end of the range

        //start

        if( (varSelectRange.startContainer == varSelectRange.endContainer &&
varSelectRange.startContainer.childNodes.length != 0)

            || (varSelectRange.startContainer ==
varSelectRange.endContainer.parentNode)){

            var varNextNode =
varSelectRange.startContainer.childNodes[varSelectRange.startOffset];

            if(varNextNode.tagName == "BR"){

                while( varNextNode != null && varNextNode.tagName == "BR"){

                        varNextNode = varNextNode.nextSibling;

                }

                if(varNextNode != null){

                    varSelectRange.setStart(varNextNode, 0);

                }

            }

        }

        if( (varSelectRange.startContainer == varSelectRange.endContainer &&
varSelectRange.startContainer.childNodes.length != 0)

            || (varSelectRange.startContainer.parentNode==
varSelectRange.endContainer) ){

            //end
```

```
                var varPreviousNode =
varSelectRange.endContainer.childNodes[varSelectRange.endOffset];

                if(varPreviousNode.tagName == "BR"){

                    while( varPreviousNode != null && varPreviousNode.tagName == "BR"){

                            varPreviousNode = varPreviousNode.previousSibling;

                    }

                    if(varPreviousNode != null){

                        varSelectRange.setEnd(varPreviousNode,
varPreviousNode.textContent.length);

                    }

                }

            }

            varSelection.removeAllRanges();

            varSelection.addRange(varSelectRange);

            var parent = varSelectRange.startContainer.parentNode;

            var varNextSibling = varSelectRange.startContainer;

            while( varNextSibling != null && varNextSibling != varSelectRange.endContainer ){

                if(varNextSibling.tagName == "A") return false;

                varNextSibling = varNextSibling.nextSibling;

            }

            return true;

        }

        return false;

}


/*function:process the text selection operation, by capturing and processing all click events.

* author: Haijun Zhai

* date: 5/18/2012
```

```
* verson: 1.0
*/
function DIVTextSelection(event){
try{
	//filter right click; if button=1 left,button=2 right
	if(event.button == 2)
		return;
	var varSelection = window.getSelection();
	if(IsValidSelection()){
		//if selected text, then tag option menu can be displayed
		varStaticOptionVisible = true;
		var varSelectRange = varSelection.getRangeAt(0);
		if(varSelectRange.startContainer == varSelectRange.endContainer){
			var sWholeText = varSelectRange.startContainer.nodeValue;
			var nStartOffset = varSelectRange.startOffset;
			var nEndOffset = varSelectRange.endOffset;
			//note: endoffset not belonging to the selection

			// filter space, for instance "he is a boy", " is a boy" is selected, the code can
filter the start space in the selection to "is a boy"
			if(!IsValidBegin(sWholeText.charAt(nStartOffset))){
				var sBegineString = sWholeText.substring(nStartOffset, nEndOffset);
				var nNewStart = FirstIndexofAlNum(sBegineString);
				varSelectRange.setStart(varSelectRange.startContainer,
nNewStart+nStartOffset);

			}
			else if(nStartOffset > 0){
```

```
                    //extend the selection to the whole complete strings, for instance "he is
a boy", "s a bo" is selected, the code bellow can extend
                    // the selection to "is a boy";

                    if(IsValidBegin(sWholeText.charAt(nStartOffset)) &&
IsValidBegin(sWholeText.charAt(nStartOffset-1))){

                        var sBegineString = sWholeText.substring(0, nStartOffset);
                        var nNewStart = LastIndexofNonAlNum(sBegineString);
                        //matched nNewStart+1, if not, 0
                        varSelectRange.setStart(varSelectRange.startContainer,
nNewStart);
                    }

                }

                /// filter space, for instance "he is a boy", "is a " is selected, the code can filter
the start space in the selection to "is a"
                if(!IsValidEnd(sWholeText.charAt(nEndOffset-1))){

                    var sBegineString = sWholeText.substring(0, nEndOffset-1);
                        var nNewEnd = LastIndexofAlNum(sBegineString);
                        varSelectRange.setEnd(varSelectRange.endContainer,
nNewEnd+1);
                }
                else if(nEndOffset < sWholeText.length){
                    //extend the selection to the whole complete strings, for instance "he is
a boy", "s a bo" is selected, the code bellow can extend
                    // the selection to "is a boy";
                    if(IsValidEnd(sWholeText.charAt(nEndOffset-1)) &&
IsValidEnd(sWholeText.charAt(nEndOffset))){
```

```
                        var sEndString = sWholeText.substring(nEndOffset);
                        var nNewEnd = FirstIndexofNonAlNum(sEndString);
                        if(nNewEnd >= 0){
                                varSelectRange.setEnd(varSelectRange.endContainer,
nEndOffset + nNewEnd);

                        }else{
                                varSelectRange.setEnd(varSelectRange.endContainer,
sWholeText.length);
                        }
                }
            }
        }else{
            var sWholeTextHead = varSelectRange.startContainer.nodeValue;
            var sWholeTextTail = varSelectRange.endContainer.nodeValue;
            var nStartOffset = varSelectRange.startOffset;
            var nEndOffset = varSelectRange.endOffset;

            //note: endoffset not belonging to the selection

            // filter space, for instance "he is a boy", " is a boy" is selected, the code can
filter the start space in the selection to "is a boy"
            if(!IsValidBegin(sWholeTextHead.charAt(nStartOffset))){
                var sBegineString = sWholeTextHead.substring(nStartOffset,
nEndOffset);
                var nNewStart = FirstIndexofAlNum(sBegineString);
                varSelectRange.setStart(varSelectRange.startContainer,
nNewStart+nStartOffset);

            }
```

```
else if(nStartOffset > 0){

        //extend the selection to the whole complete strings, for instance "he is
a boy", "s a bo" is selected, the code bellow can extend

        // the selection to "is a boy";


        if(IsValidBegin(sWholeTextHead.charAt(nStartOffset)) &&
IsValidBegin(sWholeTextHead.charAt(nStartOffset-1))){


                var sBegineString = sWholeTextHead.substring(0, nStartOffset);

                var nNewStart = LastIndexofNonAlNum(sBegineString);

                //matched nNewStart+1, if not, 0

                varSelectRange.setStart(varSelectRange.startContainer,
nNewStart);

        }


}


/// filter space, for instance "he is a boy", "is a " is selected, the code can filter
the start space in the selection to "is a"

if(!IsValidEnd(sWholeTextTail.charAt(nEndOffset-1))){


        var sBegineString = sWholeTextTail.substring(0, nEndOffset-1);

                var nNewEnd = LastIndexofAlNum(sBegineString);

                varSelectRange.setEnd(varSelectRange.endContainer,
nNewEnd+1);

}

else if(nEndOffset < sWholeTextTail.length){

        //extend the selection to the whole complete strings, for instance "he is
a boy", "s a bo" is selected, the code bellow can extend

        // the selection to "is a boy";
```

```
                                        if(IsValidEnd(sWholeTextTail.charAt(nEndOffset-1)) &&
IsValidEnd(sWholeTextTail.charAt(nEndOffset))){
                                                  var sEndString = sWholeTextTail.substring(nEndOffset);
                                                  var nNewEnd = FirstIndexofNonAlNum(sEndString);
                                                  if(nNewEnd >= 0){
                                                            varSelectRange.setEnd(varSelectRange.endContainer,
nEndOffset + nNewEnd);

                                                  }else{
                                                            varSelectRange.setEnd(varSelectRange.endContainer,
sWholeTextTail.length);
                                                  }
                                        }
                              }
                    }
                    varSelection.removeAllRanges();
                    varSelection.addRange(varSelectRange);
                    varStaticCurrentSelectRange = varSelectRange.cloneRange();
          }
}catch(e){
          alert("Invalid selection!\nPlease follow the instructions above!");
}
}



//map: saving annotation results
//reuslt format:  key: medicationname(or medicationtype)_div_id,  value:Array(startoffset-endoffset1,
startoffset-endoffset2, ...)
//id used for identifying each unique result
```

```
var varStaticCountID = 0;

function Map(){
	this.elements=new Array();
	this.size=function(){
		return this.elements.length;
	}
	this.put=function(_key,_value){
		this.elements.push({key:_key,value:_value});
	}
	this.remove=function(_key){
		var bln=false;
		try{
			for (i=0;i<this.elements.length;i++){
				if (this.elements[i].key==_key){
					this.elements.splice(i,1);
					return true;
				}
			}
		}catch(e){
			bln=false;
		}
		return bln;
	}
	this.containsKey=function(_key){

		var bln=false;
		try{
```

```
				for (i=0;i<this.elements.length;i++){
				if (this.elements[i].key==_key){
					bln=true;
				}
				}
			}catch(e){
				bln=false;
			}
			return bln;
		}
		this.get=function(_key){
			try{
				for (i=0;i<this.elements.length;i++){
					if (this.elements[i].key==_key){
						return this.elements[i];
					}
				}
			}catch(e){
			return null;
			}
		}
}

//global variable for saving tagging results
var varStaticMapResult = new Map();
```

```
//result display functions

/*

* fucntion: sort  the result, to guirantee an annotation item(formated as:startoffset-endoffset1,startoffset-endoffset2...) will be rank  by the startoffset from small number to large

* input: varItemA [String: indicating an notation Item, format[startoffset-endoffset]]

* input: varItemB [String: indicating the notations Items to be displayed in the correseponding textbox, format:[startoffset-endoffset1,startoffset-endoffset2...]]

* return: [boolean: true/false denotes startoffset-endoffset1 of varItemA less/greater than that of varItemB]

* author: Haijun Zhai

* date: 5/18/2012

* verson: 1.0

*/

function ItemOfAnnationCompare(varItemA, varItemB)

{

	var varArrayA = varItemA.split("-");

	var varArrayB = varItemB.split("-");

	var varStartOffsetA = parseInt(varArrayA[0]);

	var varStartOffsetB = parseInt(varArrayB[0]);

	return (varStartOffsetA - varStartOffsetB);

}


/*

* fucntion: sort  the result, to guirantee an annotation item(formated as:startoffset-endoffset1,startoffset-endoffset2...) will be rank  by the startoffset from small number to large

* input: varAnnotationA [array: indicating an anotation, startoffset-endoffset1,startoffset-endoffset2,...]

* input: varAnnotationB [array: indicating an anotation, startoffset-endoffset1,startoffset-endoffset2,...]

* return: [boolean: true/false denotes startoffset-endoffset1 of varAnnotationA less/greater than that of varAnnotationB]
```

```
* author: Haijun Zhai
* date: 5/18/2012
* verson: 1.0
*/
function AnnotationCompare(varAnnotationA, varAnnotationB)
{
	var varArrayA = varAnnotationA[0].split("-");
	var varArrayB = varAnnotationB[0].split("-");
	var varStartOffsetA = parseInt(varArrayA[0]);
	var varStartOffsetB = parseInt(varArrayB[0]);
	return (varStartOffsetA - varStartOffsetB);
}

/*fucntion: get the term from the original text by the offset
* input: varText[String: original text]
* input: varOffset[String: offset arguments, format: startoffset-endoffset]
* output: varTerm[String: the term indicated by varText and varOffset]
*/
function GetTextByOffsets(varText, varOffset){
	try{
		var varItems = varOffset.split("-");
		var varStartOffset = parseInt(varItems[0]);
		var varEndOffset = parseInt(varItems[1]);
		return varText.substring(varStartOffset, varEndOffset);
	}catch(err){
		//alert("Error display annotation result!\n Please contact the author and reload this page!")
		alert("Invalid selection!\nPlease follow the instructions above!");
```

```
        }

}
/*
* fucntion: get the whole text of the div, note that the br nodes replace with spaces
* input: varResultID[String, DIVID]
* author: Haijun Zhai
* date: 5/15/2012
* verson: 1.0
*/

function GetFullText(varResultID)
{
        var varWholeText = "";
        var varDIV = document.getElementById(varResultID);
        var varNextNode = varDIV.firstChild;
        //get P node
        while(varNextNode != null && varNextNode.tagName != "P"){
                varNextNode = varNextNode.nextSibling;
        }
        if(varNextNode!= null){
                //combine all the text
                var varChild = varNextNode.firstChild;
                while(varChild != null){
                        if(varChild.tagName == "BR")varWholeText += " ";
                        else varWholeText += varChild.textContent;
                        varChild = varChild.nextSibling;
                }
```

```
            }
            return varWholeText;
}


/*
* fucntion:display the annotation results in the table, note the result will be rank  by the startoffset from small number to large
* input: arrayResultTempMedName [String Array: indicating the notations to be displayed table, format: string1, string2,...,stringi[string1,string2....],...stringn,]
* input: arrayResultTempMedType [String Array: indicating the notations to be displayed table]
* input: varParentNode[Node: the parent node of this div table]
* input: varDIVIDTemp[String: identify this div table]
* input: varResultID[String: DIVID]
* author: Haijun Zhai
* date: 5/15/2012
* verson: 1.0
*/
function DispResInDIVTable(arrayResultTempMedName, arrayResultTempMedType, varParentNode, varDIVIDTemp, varResultID){
            try{

                        //create table for displaying annotation results
                        var varTableDivNode = document.createElement("DIV");
                        varTableDivNode.setAttribute("class", "divTableAnnotationRes");
                        varTableDivNode.setAttribute("id", varDIVIDTemp);
                        //title
                        var vartableTile = document.createElement("LI");
                        varTableDivNode.appendChild(vartableTile);
                        vartableTile.appendChild(document.createTextNode("Annotated entity list:"));
```

```
vartableTile.setAttribute("class", "divTableAnnotationRes_Title");
//head
/*var varTableHeadCol1 = document.createElement("LI");
varTableHeadCol1.setAttribute("class", "divTableAnnotationRes_Head_C1");
varTableDivNode.appendChild(varTableHeadCol1);
varTableHeadCol1.appendChild(document.createTextNode("Order"));
*/
var varTableHeadCol2 = document.createElement("LI");
varTableHeadCol2.setAttribute("class", "divTableAnnotationRes_Head_C2");
varTableDivNode.appendChild(varTableHeadCol2);
varTableHeadCol2.appendChild(document.createTextNode("Medication Name"));
var varTableHeadCol3 = document.createElement("LI");
varTableHeadCol3.setAttribute("class", "divTableAnnotationRes_Head_C3");
varTableDivNode.appendChild(varTableHeadCol3);
varTableHeadCol3.appendChild(document.createTextNode("Medication Type"));
//create each row
var varWholeText = GetFullText(varResultID);
for(var i = 0; (i < arrayResultTempMedName.length) ||(i <
arrayResultTempMedType.length); i++){

    /*
    var varCol1 =  document.createElement("LI");
    varCol1.setAttribute("class", "divTableAnnotationRes_Col1");
    varCol1.appendChild(document.createTextNode(i+1));
    varTableDivNode.appendChild(varCol1);
    */
    var varCol2 =  document.createElement("LI");
    varCol2.setAttribute("class", "divTableAnnotationRes_Col2");
```

```
				varTableDivNode.appendChild(varCol2);
				var varCol3 =  document.createElement("LI");
				varCol3.setAttribute("class", "divTableAnnotationRes_Col3");
				varTableDivNode.appendChild(varCol3);
				//create medication name col
				if(i < arrayResultTempMedName.length){
					var varStrTemp = "";
					for(var j = 0; j < arrayResultTempMedName[i].length; j++){
						if(j != 0) varStrTemp += " ";
						varStrTemp += GetTextByOffsets(varWholeText,
arrayResultTempMedName[i][j]);
					}
					if(varStrTemp.length > 38) varStrTemp = varStrTemp.substring(0, 34) +
"...";
					varCol2.appendChild(document.createTextNode(varStrTemp));
				}
				else{
					varCol2.appendChild(document.createTextNode("----"));
				}
				//create medication type col
				if(i < arrayResultTempMedType.length){
					var varStrTemp = "";
					for(var j = 0; j < arrayResultTempMedType[i].length; j++){
						if(j != 0) varStrTemp += " ";
						varStrTemp += GetTextByOffsets(varWholeText,
arrayResultTempMedType[i][j]);
					}
					if(varStrTemp.length > 38) varStrTemp = varStrTemp.substring(0, 34) +
"...";
```

```
                    varCol3.appendChild(document.createTextNode(varStrTemp));
                }
                else{
                    varCol3.appendChild(document.createTextNode("----"));
                }
            }
            //varParentNode.appendChild(varTableDivNode);
            varParentNode.parentNode.insertBefore(varTableDivNode,
varParentNode.nextSibling.nextSibling);
        }catch(err){
            alert("Invalid selection!\nPlease follow the instructions above!");
        }
}

/*
* fucntion: Display the annotation results in the input textbox, note that each div is identified by divid
which is the taskid_"divtable"
* input: varResultID [String: indicating the notations to be displayed in the correseponding textbox,
DivID]
* input: varType [String: Indicating the textbox to display the notations, note that this is the type,
"medicationame/medicationtype"]
* author: Haijun Zhai
* date: 5/15/2012
* verson: 1.0
*/
function DispResInTBox(varResultID, varType)
{
 try{
            var arrayResultTempMedName = new Array();
```

```
var arrayResultTempMedType = new Array();
//Sort results
for(var i = 0; i < varStaticMapResult.size(); i++){
	//match all the medicationname and medicationtype tags
	var varIDTemp = varStaticMapResult.elements[i].key;
	var varIndexTemp;
	if( (varIndexTemp = varIDTemp.indexOf("medicationname_" + varResultID)) ==
0){

		//sort a notation item
		var arrayItemResult = new Array();
		for(var j = 0; j < varStaticMapResult.elements[i].value.length; j++){

			arrayItemResult.push(varStaticMapResult.elements[i].value[j]);
		}
		arrayItemResult.sort(ItemOfAnnationCompare);
		arrayResultTempMedName.push(arrayItemResult);
	}
	else if( (varIndexTemp = varIDTemp.indexOf("medicationtype_" + varResultID))
== 0){
		//sort a notation item
		var arrayItemResult = new Array();
		for(var j = 0; j < varStaticMapResult.elements[i].value.length; j++){

			arrayItemResult.push(varStaticMapResult.elements[i].value[j]);
		}
		arrayItemResult.sort(ItemOfAnnationCompare);
		arrayResultTempMedType.push(arrayItemResult);
```

```
		}
	}
	arrayResultTempMedName.sort(AnnotationCompare);
	arrayResultTempMedType.sort(AnnotationCompare);

	var varStringResultTempMedName = "";
	var varStringResultTempMedType = "";
	//concat the strings of all the annotations of Medicationname
	for(var i = 0; i < arrayResultTempMedName.length; i++){
		var varItemResult = "[";
		for(var j = 0; j < arrayResultTempMedName[i].length; j++){
			varItemResult += arrayResultTempMedName[i][j] + ",";
		}
		varItemResult += "]";
		varStringResultTempMedName += varItemResult + ";";
	}
	//concat the strings of all the annotations of Medicationtype
	for(var i = 0; i < arrayResultTempMedType.length; i++){
		var varItemResult = "[";
		for(var j = 0; j < arrayResultTempMedType[i].length; j++){
			varItemResult += arrayResultTempMedType[i][j] + ",";
		}
		varItemResult += "]";
		varStringResultTempMedType += varItemResult + ";";
	}
	//display in the Textbox
	var varInputTextBox;
	var varInputs = document.getElementsByTagName("input");
```

```
			for(var i = 0; i < varInputs.length; i++){
				if(varInputs[i].name.indexOf(varResultID + "[" + varType + "]") >= 0){
					varInputTextBox = varInputs[i];
					if(varType == "medicationname") varInputs[i].value = 
varStringResultTempMedName;
					else if(varType == "medicationtype") varInputs[i].value = 
varStringResultTempMedType;
				}
				//display in the required input textbox medicationnamemedicationtype
				if(varInputs[i].name.indexOf(varResultID + 
"[medicationnamemedicationtype]") >= 0){
					varInputs[i].value = "";
					if(varStringResultTempMedName.length > 1)varInputs[i].value = 
"medicationname:" + varStringResultTempMedName;//.replace(/[^a-zA-Z0-9]/g,"");
					if(varStringResultTempMedType.length > 1)varInputs[i].value += 
"medicationtype:" + varStringResultTempMedType;//.replace(/[^a-zA-Z0-9]/g,"");
				}
			}



			//Display in the div Table, note that each div is identified by divid which is the 
taskid_"divtable"
			//if previously created, the remove it and creat a new one
			var varTableDivNode = document.getElementById(varResultID+"_divtable");
			if(varTableDivNode != null){
				varTableDivNode.parentNode.removeChild(varTableDivNode);
			}
			DispResInDIVTable(arrayResultTempMedName, arrayResultTempMedType, 
varInputTextBox.parentNode.parentNode, varResultID+"_divtable", varResultID);
		}catch(err){
			//alert("Html format error!\n Please contact the author and reload this page!");
```

```
            alert("Invalid selection!\nPlease follow the instructions above!");
        }
}

/*
* fucntion: Display a menu of the targe entity types
* input: div [String: indicates the DivID]
* input: menuDiv [String: indicates id assigned for this menu]
* input: menuList[Array, indicates the targe entity types]
* input: classList[Array, indicates the display configuration]
* author: Haijun Zhai
* date: 5/15/2012
* verson: 1.0
*/
function RightHandMenu(div,menuDiv,menuList,classList)
{
        var oThis = this;
        var oDivID = div;
        this._menuList =
        {

        }
        this._classList =
        {
                objClass:'',
                MenuClass:'',
                liAbleClass:'',
                liMouseOverClass:''
```

```
    }
    this.Init = function()
    {

        this._obj = $(div);
        //this._obj = document.body;
        this._obj.oncontextmenu = function(e){oThis.ShowMenu(e)};
        this._obj.className = this._classList.objClass;
        //document.onclick = function(){oThis.HiddenMenu()};
        document.onclick = function(){oThis.HiddenMenu()};
        //this._obj.onclick = function(){oThis.HiddenMenu()};
        objToObj(this._classList, classList);
        objToObj(this._menuList, menuList);
    }

    this.CreateMenu = function()
    {
        if($(menuDiv))
        {
            alert("Error!\n Please reload this page!");
            return;
        }

        this._menu = document.createElement("DIV");
        this._menu.id = menuDiv;
  this._menu.oncontextmenu = function(e){stopBubble(e)};
        this._menu.className = this._classList.MenuClass;
        this._menu.style.display = "none";
```

```
        document.body.appendChild(this._menu);

    }

    this.CreateMenuList = function()
    {
        var varDivMainMenu = document.createElement("DIV");
        varDivMainMenu.className ="cmenu";
        var varDivBackMenu = document.createElement("DIV");
        varDivBackMenu.className ="cmenuBack";
        this._menu.appendChild(varDivMainMenu);
        this._menu.appendChild(varDivBackMenu);
        for(var pro in this._menuList)
        {
            var li = document.createElement("LI");
            li.innerHTML = pro;
            varDivMainMenu.appendChild(li);
            li.className = this._classList.liAbleClass;
            li.onclick = this._menuList[pro];
            li.onmouseover =
function(){oThis.ChangeLiClass(this,oThis._classList.liMouseOverClass)}
            li.onmouseout = function(){oThis.ChangeLiClass(this,oThis._classList.liAbleClass)}
        }
    }

    this.ChangeLiClass = function(obj,name)
    {
        obj.className = name
```

```
	}

	this.ShowMenu = function(e)
	{
		if(!varStaticOptionVisible) return;
		varStaticDIVID = oDivID;
		var e = e || window.event;
		stopBubble(e);
		var offsetX = e.clientX;
		var offsetY = e.clientY;
		with(this._menu.style)
		{
   display = "block";
   top = offsetY +
(document.documentElement.scrollTop?document.documentElement.scrollTop:document.body.scrollT
op) + "px";
   left = offsetX +
(document.documentElement.scrollLeft?document.documentElement.scrollLeft:document.body.scrollL
eft) + "px";
   //alert(left "  ");

		}
		varStaticOptionVisible = false;
	}

	this.HiddenMenu = function()
	{
		//this._menu.style.display = "none";
		var varAllDiv = document.getElementsByTagName("DIV");
```

```
                    for(var i = 0; i < varAllDiv.length; i++){
                        if(varAllDiv[i].id.indexOf("TestRightMenuDivZbills") >= 0){
                            varAllDiv[i].style.display = "none";
                        }
                    }
        }
        this.Init();
        this.CreateMenu();
        this.CreateMenuList();
}
function stopBubble(oEvent)
{
        if(oEvent.stopPropagation) oEvent.stopPropagation();
        else oEvent.cancelBubble = true;
         if(oEvent.preventDefault) oEvent.preventDefault();
   else oEvent.returnValue = false;
}

function $(div)
{
        return 'string' == typeof div ? document.getElementById(div) : div;
}
function objToObj(destination,source)
{
        for(var pro in source)
        {
            destination[pro] = source[pro];
        }
```

```
	return destination;
}

/*funtion: process the action related to highlighting medication name
* author: Haijun Zhai
* date: 5/15/2012
* verson: 1.0
*/
function Name(event)
{
	//var varStaticHighlightCount = 0;
	//var for map the relations between highlight_id and resultmap_id, entry format  [highlight_id, resultmap_id]; note that resultmap_id may be repeated
	//var varStaticMapHighlightToResult = new Array();
	//note that offset should be calculated before highlighted(checked by debuging)
	var varStartOffsetTemp = CalculateStartOffset(varStaticCurrentSelectRange);
	var varEndOffsetTemp = CalculateEndOffset(varStaticCurrentSelectRange);
	var varIsAddCount = false;
	if(!event.ctrlKey) {
		varStaticCountID++;
		varIsAddCount = true;
	}
	if(HightlightRange(varStaticCurrentSelectRange, "medicationname_" + varStaticDIVID + "_"+varStaticCountID, "highlightmedicationname")){
		var varStringTemp = varStartOffsetTemp + "-" + varEndOffsetTemp;
		var varId = "medicationname_" +varStaticDIVID + "_" + varStaticCountID;
		//store and highlight the current highlighting selections
		//save highlight results
```

```
			//consideration for discontinous selection, if /or not pressing down ctrl indicates yes/no a continous selection
			if(varStaticMapResult.containsKey(varId)){
				varStaticMapResult.get(varId).value.push(varStringTemp);
			}
			else{
				//save the relations between string results and highlights
				varStaticMapResult.put(varId, new Array(varStringTemp));
			}
			//varStaticMapHighlightToResult[varStaticHighlightCount++] = varStaticCountID;
			DispResInTBox(varStaticDIVID, "medicationname");
		}else{
			if(varIsAddCount)
				varStaticCountID--;
		}
}

/*funtion: process the action related to highlighting medication type
* author: Haijun Zhai
* date: 5/15/2012
* verson: 1.0
*/
function Type(event)
{
		//var varStaticHighlightCount = 0;
		//var for map the relations between highlight_id and resultmap_id, entry format  [highlight_id, resultmap_id]; note that resultmap_id may be repeated
		//var varStaticMapHighlightToResult = new Array();
```

```
	//note that offset should be calculated before highlighted(checked by debuging)
	var varStartOffsetTemp = CalculateStartOffset(varStaticCurrentSelectRange);
	var varEndOffsetTemp = CalculateEndOffset(varStaticCurrentSelectRange);
	var varIsAddCount = false;
	if(!event.ctrlKey) {
		varStaticCountID++;
		varIsAddCount = true;
	}
	if(HightlightRange(varStaticCurrentSelectRange, "medicationtype_" + varStaticDIVID +
"_"+varStaticCountID, "highlightmedicationtype")){
		var varStringTemp = varStartOffsetTemp + "-" + varEndOffsetTemp;
		var varId = "medicationtype_" +varStaticDIVID + "_" + varStaticCountID;
		//store and highlight the current highlighting selections
		//save highlight results
		//consideration for discontinous selection, if /or not pressing down ctrl indicates yes/no
a continous selection
		if(varStaticMapResult.containsKey(varId)){
			varStaticMapResult.get(varId).value.push(varStringTemp);
		}
		else{
			//save the relations between string results and highlights
			varStaticMapResult.put(varId, new Array(varStringTemp));
		}
		//varStaticMapHighlightToResult[varStaticHighlightCount++] = varStaticCountID;
		DispResInTBox(varStaticDIVID, "medicationtype");
	}else{
		if(varIsAddCount)
			varStaticCountID--;
```

```
	}

}

var menuList =
{
	"Medication Name":Name,
	"Medication Type":Type
}
var classList =
{
	MenuClass:'cmenuAll',
	liAbleClass:'liAble',
	liMouseOverClass:'liMouseOver'
}

//get the browser name
var varBrowserName = navigator.userAgent.toLowerCase();
if( !(varBrowserName.match(/firefox/) || varBrowserName.match(/chrome/))){
	alert("This task can only run on Google Chrome and Mozilla Firefox!\n Please switch to one of them to continue this task!");
}
/*
* funtion: extend current selection by including the right nearest char
* author: Haijun Zhai
* date: 5/29/2012
* verson: 1.0
```

```
*/

function ExtendOneChar(varEvent)
{

	//varEvent.stopPropagation();
	if( IsValidSelection() ){
		var varSelection = window.getSelection();
		var varSelectRange = varSelection.getRangeAt(0);
		var sWholeText = varSelectRange.endContainer.nodeValue;
		var nStartOffset = varSelectRange.startOffset;
		var nEndOffset = varSelectRange.endOffset;
		//note: endoffset not belonging to the selection
		if(nEndOffset <= sWholeText.length - 1){
			varSelectRange.setEnd(varSelectRange.endContainer, nEndOffset+1);
		}else{
			var varNextNode = varSelectRange.endContainer.nextSibling;
			while( varNextNode != null && varNextNode.nodeType != 3){
				if(varNextNode.tagName == "BR")
					varNextNode = varNextNode.nextSibling;
				else return;
			}
			if(varNextNode != null){
				varSelectRange.setEnd(varNextNode, 1);
			}
		}
		varStaticOptionVisible = true;
		varSelection.removeAllRanges();
```

```
        varSelection.addRange(varSelectRange);
        varStaticCurrentSelectRange = varSelectRange.cloneRange();
    }

}

/*
* funtion: shrink current selection by excluding the rightest char
* author: Haijun Zhai
* date: 5/29/2012
* verson: 1.0
*/

function ShrinkOneChar(varEvent)
{
    //varEvent.stopPropagation();
    if( IsValidSelection() ){
        var varSelection = window.getSelection();
        var varSelectRange = varSelection.getRangeAt(0);
        if(varSelectRange.endContainer == varSelectRange.startContainer){
            var sWholeText = varSelectRange.endContainer.nodeValue;
            var nStartOffset = varSelectRange.startOffset;
            var nEndOffset = varSelectRange.endOffset;
            //note: endoffset not belonging to the selection
            if(nEndOffset-1>nStartOffset){
                varSelectRange.setEnd(varSelectRange.endContainer, nEndOffset-1);
            }else{
                varSelection.removeAllRanges();
```

```
                varStaticCurrentSelectRange = null;
                varStaticOptionVisible = false;
                return;
            }
        }else{//selected more than one node
            var nStartOffset = varSelectRange.startOffset;
            var nEndOffset = varSelectRange.endOffset;
            if(nEndOffset > 1)varSelectRange.setEnd(varSelectRange.endContainer,
nEndOffset-1);
            else{
                var varPreviousNode = varSelectRange.endContainer.previousSibling;

                while( varPreviousNode != null && varPreviousNode.nodeType != 3){
                    if(varPreviousNode.tagName == "BR")
                        varPreviousNode = varPreviousNode.previousSibling;
                    else return;
                }
                if(varPreviousNode != null){
                    varSelectRange.setEnd(varPreviousNode,
varPreviousNode.textContent.length);
                }
            }
        }
        varSelection.removeAllRanges();
        varSelection.addRange(varSelectRange);
        varStaticCurrentSelectRange = varSelectRange.cloneRange();
    }
}
```

```
/*
* funtion: hide the input textbox named "medicationnamemedicationtype"
* input: varDIVID[String, id of the div of the task]
* author: Haijun Zhai
* date: 5/15/2012
* verson: 1.0
*/
function HideAllRequireField(varDIVID)
{
	//get all input check boxes and hide them
	var varDIV = document.getElementById(varDivID);
	if(varDIV.lastElementChild != null){
		varDIV.lastElementChild.style.display = "none";
		var TextboxID = "[medicationnamemedicationtype]";
		var varInputs = document.getElementsByTagName("input");
		for(var i = 0; i < varInputs.length; i++){
			var nPos = varInputs[i].name.indexOf(TextboxID);
			//automatically fill this required input
			//if(nPos >= 0){
			//	varInputs[i].value = "---";
			//}
		}
	}
}

/*
* component main: this is the main function of the program, which actives all the listening functions for user annotation operations, hides some input textbox and so on.
```

```
* author: Haijun Zhai
* author: Haijun Zhai
* date: 5/15/2012
* verson: 1.0
*/
//////
{
	var TextboxID = "[medicationname]";
	var varInputs = document.getElementsByTagName("input");
	var varTempMenuCount = 0;
	for(var i = 0; i < varInputs.length; i++){
		var nPos = varInputs[i].name.indexOf(TextboxID);
		if(nPos >= 0){
			var varDivID = varInputs[i].name.substring(0, nPos);
			HideAllRequireField(varDivID);
			var varNotationMenu = new
RightHandMenu(varDivID,"TestRightMenuDivZbills" + varTempMenuCount++, menuList,classList);
			var varDIVTemp = document.getElementById(varDivID);
			varDIVTemp.addEventListener("mouseup", DIVTextSelection);// =
test;//function(){return function(){DIVTextSelection(window.event);}}();
			//generate the selection tool
			var varDIVSelectionDIV = document.createElement("DIV");
			varDIVSelectionDIV.setAttribute("class", "divSelectionTool");
			var varButtonExtend = document.createElement("button");
			//varButtonExtend.setAttribute("href", "javascript:void(0);");
			varButtonExtend.appendChild(document.createTextNode("Extend Highlight"));
			varButtonExtend.setAttribute("type", "button");
			varButtonExtend.onclick = function(){return
function(){ExtendOneChar(window.event);}}();
```

```
                varDIVSelectionDIV.appendChild(varButtonExtend);

                var varButtonShrink = document.createElement("button");

                varButtonShrink.appendChild(document.createTextNode("Shrink Highlight"));

                varButtonShrink.setAttribute("type", "button");

                varButtonShrink.onclick = function(){return
function(){ShrinkOneChar(window.event);}}();

                varDIVSelectionDIV.appendChild(varButtonShrink);

                varDIVTemp.parentNode.insertBefore(varDIVSelectionDIV,
varDIVTemp.nextSibling);


            }

        }

}




/*

* component Error_page: these codes aim to remanage the error page for turkers in an understandable way based on the CrowdFlower output, by hidding the offsets information and show the user's answer(map the offsets to text))

* an example is show in this page in html node P named "unit_message" (which is used for indicating the error massage in CF Error page)

*

*/

//these codes use for hiding the offset information and show the result user dis

var varPOffsetResult = document.getElementsByTagName('p');

for(var i = 0; i < varPOffsetResult.length; i++)

{

  //unit_message used for identify error messagepage

  if(varPOffsetResult[i].className == "unit_message"){

    var varSibling = varPOffsetResult[i].firstChild.nextSibling;
```

```
  varPOffsetResult[i].removeChild(varSibling.nextSibling.nextSibling.nextSibling);
  varPOffsetResult[i].removeChild(varSibling.nextSibling.nextSibling);
  varPOffsetResult[i].removeChild(varSibling.nextSibling);
   //offset information from the error page
   var varUserResult = varSibling.textContent;
  varPOffsetResult[i].removeChild(varSibling);
   if(varUserResult == "" || varUserResult == "nothing"){  //nothing
  varPOffsetResult[i].insertBefore(document.createElement("br"),
varPOffsetResult[i].firstChild.nextSibling);
  varPOffsetResult[i].insertBefore(document.createTextNode("nothing"),
varPOffsetResult[i].firstChild.nextSibling);
    varPOffsetResult[i].insertBefore(document.createElement("br"),
varPOffsetResult[i].firstChild.nextSibling);
   }else{
    varUserResult = varUserResult.replace(/[\]\[\"]/g, "");
    var varOffsets = varUserResult.split(";");
    //map the offset to the text
    var varTextboxIDTemp = "[medicationname]";
    var varInputs = document.getElementsByTagName("input");
    var varTempMenuCount = 0;
    for(var j = 0; j < varInputs.length; j++){
     var nPos = varInputs[j].name.indexOf(varTextboxIDTemp);
     if(nPos >= 0){
      var varDivID = varInputs[j].name.substring(0, nPos);
      var varAllOriginalText = GetFullText(varDivID);  //get the original full text from div

      for(var k = varOffsets.length-1; k >= 0; k--){
       if(varOffsets[k].length > 2){
        var varOffsetItems = varOffsets[k].split(",");
```

```
            var varEntityTokens = "";
            for(var m = 0; m < varOffsetItems.length; m++){
              if( varOffsetItems[m].length > 2) varEntityTokens = varEntityTokens + " " +
GetTextByOffsets(varAllOriginalText, varOffsetItems[m]);
            }
            if(varEntityTokens.length > 0){
              var varSpan = document.createElement("SPAN");
              varSpan.style.backgroundColor="red";
              varSpan.appendChild(document.createTextNode(varEntityTokens));
              varPOffsetResult[i].insertBefore(varSpan, varPOffsetResult[i].firstChild.nextSibling);
              varPOffsetResult[i].insertBefore(document.createElement("br"),
varPOffsetResult[i].firstChild.nextSibling);
            }
          }
        }
      }
    }
  }
 }
}
/*
* funtion: control submit action
* author: Haijun Zhai
* date: 6/22/2012
* verson: 1.0
*/
function SubmitButtonClick()
{
```

```
    var varInputsTemps = document.getElementsByTagName("input");
    for(var i = 0; i < varInputs.length; i++){
        if(varInputs[i].name.indexOf("[medicationnamemedicationtype]") >= 0){
            if(varInputs[i].value.length < 1){
                alert("At least one entity should be highlighted!");
                return;
            }
        }
    }
    var varForms = document.getElementsByTagName("form");
    varForms[0].submit();
}
//change response of the submit button to our function
 {
    var varInputsTemps = document.getElementsByTagName("input");
    for(var i = 0; i < varInputs.length; i++){

        if(varInputs[i].className == "submit"){
            varInputs[i].setAttribute("type", "button");
            varInputs[i].onclick = function(){return function(){SubmitButtonClick();}}();

        }
    }
}
/*
* funtion: find what child node contain this offset.
* input: varParent[Node, parent node <p>]
* input: varOffset[int, indicate the offset]
```

```
* input: varIsStart[bool, indicate this is a start of an offset]

* output: varOffsetInChild[int, indicate the offset in this child]

* output: varChildNode[Node, Child node <text>, indicate the child contians the offset]

* author: Haijun Zhai

* date: 7/2/2012

* verson: 1.0

*/

function LocateChild(varParent, varOffset, varIsStart){

	var varChildNode;

	var varChild = varParent.firstChild;

	var varTotalOffset = 0;

	var varOffsetInChild = 0;

	while(varChild){

		if(varChild.tagName == "BR") varTotalOffset++;

		else if(varChild.nodeType != 3) varTotalOffset+= varChild.textContent.length;

		else{//text node

			if(varIsStart){

				if(varTotalOffset + varChild.textContent.length > varOffset){

					varOffsetInChild = (varOffset-varTotalOffset);

					varChildNode = varChild;

					break;

				}else varTotalOffset+= varChild.textContent.length;

			}else{

				if(varTotalOffset + varChild.textContent.length >= varOffset){

						varOffsetInChild = (varOffset-varTotalOffset);

						varChildNode = varChild;

						break;

				}else varTotalOffset+= varChild.textContent.length;
```

```
                }
            }
            varChild = varChild.nextSibling;
        }
        return [varOffsetInChild, varChildNode];
}



/*
* funtion find the node by name
* input: varTag[String, for p, input]
* input: varName[String, indicate the name of the target node]
* output: varNode[Node, ]
* author: Haijun Zhai
* date: 7/2/2012
* verson: 1.0
*/
function GetElementByName(varTag, varName)
{
        var varNodes = document.getElementsByTagName(varTag);
        for(var i = 0; i < varNodes.length; i++){
            var nPos = varNodes[i].name.indexOf(varName);
            if(nPos >= 0){
                return varNodes[i];
            }
        }
}
```

```
/*

* component Preannotation_load: load preannotation information (indicated by offsets) and highlight corresponding strings, the format of the info is format:[startoffset-endoffset1,startoffset-endoffset2,...,];[startoffset-endoffset1,startoffset-endoffset2,...,]

*

*/

{

	var TextboxID = "[medicationname]";

	var varInputs = document.getElementsByTagName("input");

	var varTempMenuCount = 0;

	for(var i = 0; i < varInputs.length; i++){

		var nPos = varInputs[i].name.indexOf(TextboxID);

		if(nPos >= 0){

			var varDivID = varInputs[i].name.substring(0, nPos);

			var varDIVNodeTemp = document.getElementById(varDivID);

			//if preannotation info for medicationname is not empty, high

			if(varInputs[i].value.length > 1){

				if( varInputs[i].value == "No data available" ){

					varInputs[i].value = "";

				}else{

				//MedPreannotation info

				var varMedPreannotationInfo = varInputs[i].value;

				var varMedPreInfoOffsets = varMedPreannotationInfo.replace(/[\]\[]/g,
"").split(";");

				//process each entity MedicationName

				for(var j = 0; j < varMedPreInfoOffsets.length; j++){

					varStaticCountID++;

					//process each words in entity
```

```
                                        var varMedPreInfoItems = varMedPreInfoOffsets[j].split(",");
                                        for(var k = 0; k < varMedPreInfoItems.length; k++){
                                                if(varMedPreInfoItems[k].length > 3){
                                                        varStaticCurrentSelectRange =
document.createRange();
                                                        var varStart =
parseInt(varMedPreInfoItems[k].split("-")[0]);
                                                        var varEnd =
parseInt(varMedPreInfoItems[k].split("-")[1]);
                                                        var varOffsetStartInfo =
LocateChild(varDIVNodeTemp.firstChild, varStart, true);
                                                        var varOffsetEndInfo =
LocateChild(varDIVNodeTemp.firstChild, varEnd, false);

        varStaticCurrentSelectRange.setStart(varOffsetStartInfo[1], varOffsetStartInfo[0]);

        varStaticCurrentSelectRange.setEnd(varOffsetEndInfo[1], varOffsetEndInfo[0]);
                                                        var varStringTemp = varStart + "-" + varEnd;
                                                        var varId = "medicationname_" +varDivID + "_"
+ varStaticCountID;
                                                        if(varStaticMapResult.containsKey(varId)){

        varStaticMapResult.get(varId).value.push(varStringTemp);
                                                        }
                                                        else{
                                                                //save the relations between string
results and highlights
                                                                varStaticMapResult.put(varId, new
Array(varStringTemp));
                                                        }
                                                        HightlightRange(varStaticCurrentSelectRange,
"medicationname_" + varDivID + "_"+varStaticCountID, "highlightmedicationname");
                                                }
```

```
                    }
                }
                DispResInTBox(varDivID, "medicationname");
                }
            }
            var varMedTypeInput = GetElementByName("input", varDivID +
"[medicationtype]");
            var varMedTypePreannotationInfo = varMedTypeInput.value;
            if(varMedTypePreannotationInfo.length > 1 ){
                if(varMedTypePreannotationInfo == "No data available"){
                    varMedTypeInput.value = "";
                }else{
                var varMedTypePreInfoOffsets =
varMedTypePreannotationInfo.replace(/[\]\[]/g, "").split(";");
                //process each entity MedicationType
                for(var j = 0; j < varMedTypePreInfoOffsets.length; j++){
                    varStaticCountID++;
                    //process each words in entity
                    var varMedPreInfoItems =
varMedTypePreInfoOffsets[j].split(",");
                    for(var k = 0; k < varMedPreInfoItems.length; k++){
                        if(varMedPreInfoItems[k].length > 3){
                            varStaticCurrentSelectRange =
document.createRange();
                            var varChildNode;
                            var varStart =
parseInt(varMedPreInfoItems[k].split("-")[0]);
                            var varEnd =
parseInt(varMedPreInfoItems[k].split("-")[1]);
                            var varOffsetStartInfo =
LocateChild(varDIVNodeTemp.firstChild, varStart, true);
```

```
}

                                        var varOffsetEndInfo =
LocateChild(varDIVNodeTemp.firstChild, varEnd, false);

    varStaticCurrentSelectRange.setStart(varOffsetStartInfo[1], varOffsetStartInfo[0]);

    varStaticCurrentSelectRange.setEnd(varOffsetEndInfo[1], varOffsetEndInfo[0]);
                                        var varStringTemp = varStart + "-" + varEnd;
                                        var varId = "medicationtype_" +varDivID + "_" +
varStaticCountID;
                                        if(varStaticMapResult.containsKey(varId)){

    varStaticMapResult.get(varId).value.push(varStringTemp);
                                        }
                                        else{
                                            //save the relations between string
results and highlights
                                            varStaticMapResult.put(varId, new
Array(varStringTemp));
                                        }
                                        HightlightRange(varStaticCurrentSelectRange,
"medicationtype_" + varDivID + "_"+varStaticCountID, "highlightmedicationtype");
                                    }
                                }
                            }
                            DispResInTBox(varDivID, "medicationtype");
                        }
                        }

                }
        }
}
```

```
/*

* funtion for changing the visibility of contents of the instructions

* input: content id

* author: Haijun Zhai

* date: 6/15/2012

* verson: 1.0

*/

//function CCHMCShowDetail(varInstructionID)

//{

//{var varInstructionElement =
document.getElementById('CCHMC1');if(varInstructionElement){if(varInstructionElement.style.display
== 'none') varInstructionElement.style.display = 'inline';else varInstructionElement.style.display = 'none';}

//          var varInstructionElement =
document.getElementById(varInstructionID);if(varInstructionElement){if(varInstructionElement.style.dis
play == "none") varInstructionElement.style.display = "inline";else varInstructionElement.style.display =
"none"; }

//}



//var varNotationMenu = new RightHandMenu("vart","testDiv", menuList,classList);

</script>
```

2. GUI code for linking

```
<!--

################################

#Main Developer: Haijun Zhai, PhD


# Contributor: Todd Lingren, MA

# Supervised by: Imre Solti, MD, PhD, MA
```

```
# Contact: Haijun.Zhai@cchmc.org
# Open Source License: Apache 2.0
#
# Date: 08/01/2012
# Created: 08/01/2012
# Modified: 08/01/2012 (Imre Solti)
#
##############################
# License
#######################
Copyright 2012 Imre Solti
Licensed under the Apache License, Version 2.0 (the "License");
you may not use this file except in compliance with the License.
You may obtain a copy of the License at
http://www.apache.org/licenses/LICENSE-2.0
Unless required by applicable law or agreed to in writing, software
distributed under the License is distributed on an "AS IS" BASIS,
WITHOUT WARRANTIES OR CONDITIONS OF ANY KIND, either express or implied.
See the License for the specific language governing permissions and
limitations under the License.
-->

<html><style type="text/css" media="screen">

.highlightSpanEntity {
background-color: yellow;
```

```
cursor:pointer;
 }

 .highlightSpanAttr {
background-color: lightgray;
cursor:pointer;
 }
 .divTableAnnotationRes
{
	float:top;
	width:579px;
	height: 30px;
	border-left:#999999 1px solid;
	border-top:#999999 1px solid;
	display:table;
}
.divTableAnnotationRes_Title
{
	float:left;
	width:578px;
	height: 30px;
	list-style:none outside;
	border-bottom:#999999 1px solid;
	border-right:#999999 1px solid;

	text-align:center;
```

```
	font:border;
	list-style:none;
	font-weight:900;
}
.divTableAnnotationRes_Head_C1
{
	float:left;
	width:79px;
	height: 30px;
	border-right:#999999 1px solid;
	border-bottom:#999999 1px solid;
	font:border;
	text-align:center;
	list-style:none;
	font-weight:700;
}
.divTableAnnotationRes_Head_C2
{
	float:left;
	width:248px;
	height: 30px;
	border-right:#999999 1px solid;
	border-bottom:#999999 1px solid;
	font:border;
	text-align:center;
	list-style:none;
```

```
	font-weight:700;
}
.divTableAnnotationRes_Head_C3
{
	float:left;
	width:249px;
	height: 30px;
	border-right:#999999 1px solid;
	border-bottom:#999999 1px solid;
	font:border;
	text-align:center;
	list-style:none;
	font-weight:700;
}
.divTableAnnotationRes_Col1
{
	float:left;
	width:79px;
	height: 30px;
	border-right:#999999 1px solid;
	border-bottom:#999999 1px solid;
	font:border;
	text-align:left;
	list-style:none;

}
```

```css
.divTableAnnotationRes_Col2
{
	float:left;
	width:248px;
	height: 30px;
	border-right:#999999 1px solid;
	border-bottom:#999999 1px solid;
	font:border;
	text-align:left;
	list-style:none;
	background-color:yellow;
}
.divTableAnnotationRes_Col3
{
	float:left;
	float:left;
	width:249px;
	height: 30px;
	border-right:#999999 1px solid;
	border-bottom:#999999 1px solid;
	font:border;
	text-align:left;
	list-style:none;
	background-color:lightgray;
}
```

```
</style>

<body>

<p class="unit_message">

For the question titled "linkinginfo" you answered: <span style="font-weight:bold;color:#792422;">nothing</span> but the correct answer was: <span style="font-weight:bold;color:#105b1d;">"[260-263,];[247-252,]"</span>. <br/><br/>The reason for this is: <b>"<br><span style="background-color:yellow">MTX </span>   <span style="background-color:lightgray">start </span><br><span style="background-color:yellow">MTX </span>   <span style="background-color:lightgray">infusion </span><br>"</b>

</p>

 <div class="cml jsawesome" id="u0070226170164122163172143242189148175199238074165164191045097069132215028188180137225017070063133"><p>sampling_method: Non-Probability Sample<br><br>criteria: Inclusion Criteria:<br><br>- 5-18 years of age<br><br>- Prior allogeneic Stem Cell Transplant, with any graft source, donor type, and <A name="zhj_span_1" id="zhj_span_1---yellow===red===[173-189,]===GVHD prophylaxis ===entity" class="highlightSpanEntity">GVHD prophylaxis</a> allowed<br><br>- Clinical diagnosis of cGVHD<br><br>- Need for systemic treatment, defined as <A name="zhj_span_10000" id="zhj_span_10000---lightgray===red===[272-275,]===any ===attribute" class="highlightSpanAttr">any</a> <A name="zhj_span_2" id="zhj_span_2---yellow===red===[276-286,]===medication ===entity" class="highlightSpanEntity">medication</a> or intervention delivered<br><br>- No evidence of primary disease relapse<br><br>- Signed, informed consent, and if applicable, adolescent assent<br><br>Exclusion Criteria:<br><br>- Inability to give signed informed consent <br><br>gender: Both<br><br>minimum_age: 5 Years<br><br>maximum_age: 18 Years<br><br>healthy_volunteers: No<br>mesh_term: Graft vs Host Disease<br></p>

<div class="text cml_field"><label class="legend">medication or type</label>

<div class="cml_row"><input name="u0070226170164122163172143242189148175199238074165164191045097069132215028188180137225017070063133[medication_or_type]" type="text" value="" class="medication_or_type" /></div>

</div>

  <div class="text cml_field"><label class="legend">attribute</label>
```

```
<div class="cml_row"><input
name="u007022617016412216317214324218914817519923807416516419104509706913221
5028188180137225017070063133[attribute]" type="text" value="" class="attribute" /></div>


</div>

  <div class="hidden cml_field"><input
name="u007022617016412216317214324218914817519923807416516419104509706913221
5028188180137225017070063133[linkinginfo]" type="hidden" value="" class="linkinginfo"
/></div>

  <div class="text cml_field"><label class="legend">requirearea</label>

<div class="cml_row"><input
name="u007022617016412216317214324218914817519923807416516419104509706913221
5028188180137225017070063133[requirearea]" type="text" value="---" class="requirearea
validates-required default" /></div>


</div>




  </body>

</html>


<script type="text/javascript">


function Map(){

    this.elements=new Array();

    this.size=function(){

      return this.elements.length;

    }

    this.put=function(_key,_value){
```

```
  this.elements.push({key:_key,value:_value});
}
this.remove=function(_key){
  var bln=false;
  try{
    for (i=0;i<this.elements.length;i++){
      if (this.elements[i].key==_key){
        this.elements.splice(i,1);
        return true;
      }
    }
  }catch(e){
    bln=false;
  }
  return bln;
}
this.containsKey=function(_key){

  var bln=false;
  try{
    for (i=0;i<this.elements.length;i++){
    if (this.elements[i].key==_key){
      bln=true;
    }
    }
  }catch(e){
```

```
      bln=false;
    }
    return bln;
  }
  this.get=function(_key){
    try{
    for (i=0;i<this.elements.length;i++){
      if (this.elements[i].key==_key){
        return this.elements[i];
      }
    }
    }catch(e){
    return null;
  }
  }
}

//global variable for saving Linking results
//format:  pair<[offset-entity,];[offset-attribute,]====entity----attribute ,1>
var varStaticMapResult = new Map();
//The DIV ID of this job
var varStaticDIVID = "";
//save current selected entity offset

var varStaticEntityOffset = "";
 //save current selected attribute offset
```

```
 var varStaticAttributeOffset = "";


/*
* funtion: find the html node by name
* input: varTag[String, e.g., "p", "input"]
* input: varName[String, indicates the name of the target node]
* output: varNode[Node, html node]
* author: Haijun Zhai
* date: 7/2/2012
* verson: 1.0
*/
function GetElementByName(varTag, varName)
{
  var varNodes = document.getElementsByTagName(varTag);
  for(var i = 0; i < varNodes.length; i++){
    var nPos = varNodes[i].name.indexOf(varName);
    if(nPos >= 0){
      return varNodes[i];
    }
  }
}


/*
* fucntion: remove an annotated linking
* input: varKey[string, indicates a linking information, format: [startoffset-endoffset1[entity],...,];[startoffset-endoffset2[attribute]]====entity----attribute]
* author: Haijun Zhai
```

```
* date: 7/11/2012

* verson: 1.0

*/

 function RemoveLink(varKey)

 {

  var varConfirmRes = confirm("Are you sure to remove this linkage?");

  if (varConfirmRes == true){

    varStaticMapResult.remove(varKey);

    //alert(varKey);

    DispResInTBox();

  }

 }


/*

* fucntion: display the Linking results in the table, note the result will be ranked by the startoffset of entities from small number to large, if the  startoffsets are the same then further sorting by the offsets of attribute

* input: arrayLinkResult[string array, linking informatio, format: [startoffset-endoffset1[entity],...,];[startoffset-endoffset2[attribute]]====entity----attribute,...,]

* author: Haijun Zhai

* date: 7/11/2012

* verson: 1.0

*/

function DispResInDIVTable(arrayLinkResult){

  try{


    //create table for displaying annotation results
```

```
var varTableDivNode = document.createElement("DIV");
varTableDivNode.className ="divTableAnnotationRes";
varTableDivNode.id = varStaticDIVID+"_divtable";
//title
var vartableTile = document.createElement("LI");
varTableDivNode.appendChild(vartableTile);
vartableTile.appendChild(document.createTextNode("Linking information list:"));
vartableTile.className = "divTableAnnotationRes_Title";
//head
var varTableHeadCol1 = document.createElement("LI");
varTableHeadCol1.className =  "divTableAnnotationRes_Head_C1";
varTableDivNode.appendChild(varTableHeadCol1);
varTableHeadCol1.appendChild(document.createTextNode("Remove"));

var varTableHeadCol2 = document.createElement("LI");
varTableHeadCol2.className = "divTableAnnotationRes_Head_C2";
varTableDivNode.appendChild(varTableHeadCol2);
varTableHeadCol2.appendChild(document.createTextNode("Medication or Type"));
var varTableHeadCol3 = document.createElement("LI");
varTableHeadCol3.className = "divTableAnnotationRes_Head_C3";
varTableDivNode.appendChild(varTableHeadCol3);
varTableHeadCol3.appendChild(document.createTextNode("Attribute"));
//create each row
for(var i = 0; (i < arrayLinkResult.length); i++){
  var varAllFields = arrayLinkResult[i].split("====");
  var varItemsTemp = varAllFields[1].split("----");
```

```
var varEntityTemp = varItemsTemp[0];
var varAttributeTemp = varItemsTemp[1];

var varButtonRemove = document.createElement("button");
varButtonRemove.appendChild(document.createTextNode("Remove"));
varButtonRemove.setAttribute("type", "button");
var sKeyTemp = arrayLinkResult[i];
varButtonRemove.onclick = function(sKeyTemp){return
function(){RemoveLink(sKeyTemp);}}(sKeyTemp);

var varCol1 =  document.createElement("LI");
varCol1.className = "divTableAnnotationRes_Col1";
varCol1.appendChild(varButtonRemove);
varTableDivNode.appendChild(varCol1);

var varCol2 =  document.createElement("LI");
varCol2.className = "divTableAnnotationRes_Col2";
varTableDivNode.appendChild(varCol2);
var varCol3 =  document.createElement("LI");
varCol3.className = "divTableAnnotationRes_Col3";
varTableDivNode.appendChild(varCol3);

//create entity col
if(varEntityTemp.length > 38) varEntityTemp = varEntityTemp.substring(0, 34) + "...";
varCol2.appendChild(document.createTextNode(varEntityTemp));

//create attribute col
```

```
      if(varAttributeTemp.length > 38) varAttributeTemp = varAttributeTemp.substring(0, 34) +
"...";

      varCol3.appendChild(document.createTextNode(varAttributeTemp));



    }


    var varDIVTemp = document.getElementById(varStaticDIVID);

    varDIVTemp.appendChild(varTableDivNode);

  }catch(err){

    alert("Invalid selection!\nPlease follow the instructions above!");

  }

}


/*

* fucntion: sort  the Linking result, to ensure an annotation item(formated as:[startoffset-endoffset1[entity],...,];[startoffset-endoffset2[attribute]]====entity----attribute,...,) will be rank by the startoffset of the entity and attribute from small number to large

* input: varLinkA [String: indicating a Linkage, startoffset-endoffset1[entity],...,];[startoffset-endoffset2[attribute]]====entity----attribute,...,]

* input: varLinkB [array: indicating an anotation, startoffset-endoffset1[entity],...,];[startoffset-endoffset2[attribute]]====entity----attribute,...,]

* return: [boolean: true/false denotes startoffset-endoffset1 of varLinkA less/greater than that of varLinkB]

* author: Haijun Zhai

* date: 7/11/2012

* verson: 1.0

*/

function LinkageCompare(varLinkA, varLinkB)
```

```
{
  var varLinkANew = varLinkA.split("====")[0].replace(/[\]\[]/g, "");
  var varLinkBNew = varLinkB.split("====")[0].replace(/[\]\[]/g, "");
  var varAItems = varLinkANew.split(";");
  var varBItems = varLinkBNew.split(";");
  var varAEntity = varAItems[0];
  var varAAttr = varAItems[1];
  var varAEntityStart = parseInt(varAEntity.split("-")[0]);
  var varAAttrStart = parseInt(varAAttr.split("-")[0]);

  var varBEntity = varBItems[0];
  var varBAttr = varBItems[1];
  var varBEntityStart = parseInt(varBEntity.split("-")[0]);
  var varBAttrStart = parseInt(varBAttr.split("-")[0]);
  if(varAEntityStart != varBEntityStart){
    return (varAEntityStart - varBEntityStart);
  }else{
    return (varAAttrStart - varBAttrStart)
  }
}

/*
* fucntion: Display the Linking results in linkinginfo input[String, format:[startoffset(entity)-endoffset,...,];[startoffset(attribute)-endoffset,...,]L,...,[startoffset(entity)-endoffset,...,];[startoffset(attribute)-endoffset,...,]L],note that "L" is the delimiter of linking infor, ";" is the delimiter of entity and attribute and "," is the delimiter of offsets of tokens in each entity/attribute
* author: Haijun Zhai
```

```
* date: 7/11/2012
* verson: 1.0
*/
function DispResInTBox()
{
 try{
    var arrayLinkResult = new Array();
    //Sort results
    for(var i = 0; i < varStaticMapResult.size(); i++){
      arrayLinkResult.push(varStaticMapResult.elements[i].key);
    }
    arrayLinkResult.sort(LinkageCompare);

    var varOffsetStringResultTemp = "";
    var varStringResultTemp = "";
    //concatenate the strings of all the linking info
    for(var i = 0; i < arrayLinkResult.length; i++){
      var varItemsTemp = arrayLinkResult[i].split("====");
      var varOffset = varItemsTemp[0];
      varOffsetStringResultTemp += varOffset + "L";
    }
    var varInputLinkInfoTemp = GetElementByName("input", varStaticDIVID + "[linkinginfo]");
    varInputLinkInfoTemp.value = varOffsetStringResultTemp;
    var varTableDivNode = document.getElementById(varStaticDIVID+"_divtable");
    if(varTableDivNode != null){
      varTableDivNode.parentNode.removeChild(varTableDivNode);
```

```
    }
    DispResInDIVTable(arrayLinkResult);
  }catch(err){
    alert("some errors appeared, please contact the author and reload this page!");
  }
}

/*
* function: change the bgcolor of node "A" to target color
* input: varSpanName[String, indicates the target node "A" to be changed
* input: varBackgroundColor[String, indicates target color to be set]
*/
 function SetBGColor(varSpanName, varBackgroundColor)
 {
        //flash each the spans of the entity
  var varANodes = document.getElementsByTagName("A");
  for(var i = 0; i < varANodes.length; i++){
        if(varANodes[i].name == varSpanName){
              varANodes[i].style.backgroundColor = varBackgroundColor;
        }
  }
 }
/*
* function: main funciton responses the linking action by capturing and process clicks on pre-highlighted entities
* input: varOffset[String, indicates the offset of entities/attributes, format:[startoffset-endoffset1, startoffset-endoffset2, ...,]
```

```
* input: varString[String, the text of entities/attributes]

* input: varType[String, indicates this is an entity or attribute]

*/

 function LinkAction(varOffset, varString, varType, varSpanIds)

 {

  var varInputEntityTemp = GetElementByName("input", varStaticDIVID +
"[medication_or_type]");

  var varInputAttrTemp = GetElementByName("input", varStaticDIVID + "[attribute]");


  //Main process

  if(varString.length > 0){

    if(varType == "entity"){

      varInputEntityTemp.value = varString;

      varStaticEntityOffset = varOffset;

    }else{

      varInputAttrTemp.value = varString;

      varStaticAttributeOffset = varOffset;

    }

    //A new linking is created once the two inputbox are filled

    if(varStaticEntityOffset.length > 2 && varStaticAttributeOffset.length > 2){

      var varKey = varStaticEntityOffset + ";" + varStaticAttributeOffset + "====" +
varInputEntityTemp.value + "----" + varInputAttrTemp.value;

      if(!varStaticMapResult.containsKey(varKey)){

        varStaticMapResult.put(varKey, "1");

        DispResInTBox();

      }

      varInputEntityTemp.value = "";
```

```
      varInputAttrTemp.value = "";
      varStaticEntityOffset = "";
      varStaticAttributeOffset = "";
    }
  }
 }



//
/*
* component: the main function of this program, which actives all the functions listening user annotation operations, hides some input textbox and so on.
* author: Haijun Zhai
* date: 7/11/2012
* verson: 1.0
*/
 //////
 {
  var TextboxID = "[linkinginfo]";
  var varInputs = document.getElementsByTagName("input");
  var varTempMenuCount = 0;
  for(var i = 0; i < varInputs.length; i++){
    var nPos = varInputs[i].name.indexOf(TextboxID);
    if(nPos >= 0){
      var varDivID = varInputs[i].name.substring(0, nPos);
      varStaticDIVID = varDivID;
      //hide the required input box
```

```
    //HideAllRequireField(varStaticDIVID);

    var varInputRequired = GetElementByName("input", varStaticDIVID + "[requirearea]");

    varInputRequired.parentNode.parentNode.style.display = "none";

    //set the input as readonly

    var varInputEntityTemp = GetElementByName("input", varStaticDIVID +
"[medication_or_type]");

    varInputEntityTemp.setAttribute("readOnly", "true");

    var varInputAttrTemp = GetElementByName("input", varStaticDIVID + "[attribute]");

    varInputAttrTemp.setAttribute("readOnly", "true");

    //add event to all the annotated spans

        var varAsTemp = document.getElementsByTagName("A");

        for(var k = 0; k < varAsTemp.length; k++){

            var varAName = varAsTemp[k].name;

            if(varAName.indexOf("zhj_span")>=0){

                varAsTemp[k].setAttribute("href", "javascript:void(0);");

                //note that the id field contains the arguments format: name+"---
"+arg1+"==="+arg2+...

                //id="zhj_span_2---yellow===red===[12-20,]=== investigational
excipients===attribute"

                var varAItems =  varAsTemp[k].id.split("---");

                var varAArguments = varAItems[1].split("===");

                var varOriginalColor = varAArguments[0];

                var varMoverOverColor = varAArguments[1];

                var varOffset = varAArguments[2];

                var varString = varAArguments[3];

                var varType = varAArguments[4];

                varAsTemp[k].onclick = function(varOffset,varString,varType){return
function(){LinkAction(varOffset,varString,varType);}}(varOffset,varString,varType);
```

```
}

                                    varAsTemp[k].onmouseover = function(varAName, 
varMoverOverColor){return function(){SetBGColor(varAName, 
varMoverOverColor);}}(varAName, varMoverOverColor);

                                    varAsTemp[k].onmouseout = function(varAName, 
varOriginalColor){return function(){SetBGColor(varAName, varOriginalColor);}}(varAName, 
varOriginalColor);

                        }

              }

    }

  }

}

/*

* fucntion: get the whole text of the div, note that the br nodes replace with spaces

* input: varResultID[String, DIVID]

* author: Haijun Zhai

* date: 5/15/2012

* verson: 1.0

*/


function GetFullText(varResultID)

{

            var varWholeText = "";

            var varDIV = document.getElementById(varResultID);

            var varNextNode = varDIV.firstChild;

            //get P node

            while(varNextNode != null && varNextNode.tagName != "P"){

                        varNextNode = varNextNode.nextSibling;

            }
```

```
	if(varNextNode!= null){
		//combine all the text
		var varChild = varNextNode.firstChild;
		while(varChild != null){
			if(varChild.tagName == "BR")varWholeText += " ";
			else varWholeText += varChild.textContent;
			varChild = varChild.nextSibling;
		}
	}
	return varWholeText;
}

/*fucntion: get the term from the original text by the offset
* input: varText[String: original text]
* input: varOffset[String: offset arguments, format: startoffset-endoffset]
* output: varTerm[String: the term indicated by varText and varOffset]
*/
function GetTextByOffsets(varText, varOffset){
	try{
		var varItems = varOffset.split("-");
		var varStartOffset = parseInt(varItems[0]);
		var varEndOffset = parseInt(varItems[1]);
		return varText.substring(varStartOffset, varEndOffset);
	}catch(err){
		//alert("Error display annotation result!\n Please contact the author and reload this page!")
		alert("An error appeared. Please contact the author and reload this page");
```

```
        }


}
/*
* component: these codes aim to remanage the error page for turkers in an understandable way based on the CrowdFlower output, by hiding the offsets information and show the user's answer(map the offsets to text))
* an example is show in this page in html node P named "unit_message" (which is used for indicating the error massage in CF Error page)
*
*/


//these codes use for hiding the offset information and show the result user dis
var varPOffsetResult = document.getElementsByTagName('p');
for(var i = 0; i < varPOffsetResult.length; i++)
{
  //unit_message used for identifying error messagepage
  if(varPOffsetResult[i].className == "unit_message"){
    var varSibling = varPOffsetResult[i].firstChild.nextSibling;
  varPOffsetResult[i].removeChild(varSibling.nextSibling.nextSibling.nextSibling);
  varPOffsetResult[i].removeChild(varSibling.nextSibling.nextSibling);
  varPOffsetResult[i].removeChild(varSibling.nextSibling);
    //offset information from the error page
    var varUserResult = varSibling.textContent;
  varPOffsetResult[i].removeChild(varSibling);
    if(varUserResult == "" || varUserResult == "nothing"){  //nothing
  varPOffsetResult[i].insertBefore(document.createElement("br"),
varPOffsetResult[i].firstChild.nextSibling);
```

```
  varPOffsetResult[i].insertBefore(document.createTextNode("nothing"),
varPOffsetResult[i].firstChild.nextSibling);

      varPOffsetResult[i].insertBefore(document.createElement("br"),
varPOffsetResult[i].firstChild.nextSibling);

    }else{

      varUserResult = varUserResult.replace(/[\]\[\"]/g, "");

      var varOffsetPairs = varUserResult.split("L");

      //map the offset to the text

      var varTextboxIDTemp = "[linkinginfo]";

      var varInputs = document.getElementsByTagName("input");

      var varTempMenuCount = 0;

      for(var j = 0; j < varInputs.length; j++){

        var nPos = varInputs[j].name.indexOf(varTextboxIDTemp);

        if(nPos >= 0){

          var varDivID = varInputs[j].name.substring(0, nPos);

          var varAllOriginalText = GetFullText(varDivID);  //get the original full text from div


          for(var k = varOffsetPairs.length-1; k >= 0; k--){

            if(varOffsetPairs[k].length > 2){

              var varEntity = varOffsetPairs[k].split(";")[0];

                                    var varAttribute = varOffsetPairs[k].split(";")[1];

                                    var varEntityOffset = varEntity.split(",");

                                    var varEntityTokens = "";

                                    var varAttributeOffset = varAttribute.split(",");

                                    var varAttributeTokens = "";

                                    //concatenate the text of mdeication

              for(var m = 0; m < varEntityOffset.length; m++){
```

```
               if( varEntityOffset[m].length > 2) varEntityTokens = varEntityTokens + " " +
GetTextByOffsets(varAllOriginalText, varEntityOffset[m]);

             }

                                   //concatenate the text of attribute

                                   for(var m = 0; m < varAttributeOffset.length; m++){

               if( varAttributeOffset[m].length > 2) varAttributeTokens = varAttributeTokens + " " +
GetTextByOffsets(varAllOriginalText, varAttributeOffset[m]);

             }

             if(varEntityTokens.length > 0){

                                        var varSpanAttr = document.createElement("SPAN");

               varSpanAttr.style.backgroundColor="lightgray";

               varSpanAttr.appendChild(document.createTextNode(varAttributeTokens));

               varPOffsetResult[i].insertBefore(varSpanAttr,  varPOffsetResult[i].firstChild.nextSibling);

               var varSpanEntity = document.createElement("SPAN");

               varSpanEntity.style.backgroundColor="yellow";

               varSpanEntity.appendChild(document.createTextNode(varEntityTokens));

               varPOffsetResult[i].insertBefore(varSpanEntity,
varPOffsetResult[i].firstChild.nextSibling);

               varPOffsetResult[i].insertBefore(document.createElement("br"),
varPOffsetResult[i].firstChild.nextSibling);

             }

            }

           }

          }

         }

        }

       }

}
```

</script>
