# Supplementary material for: Web 2.0-Based Crowdsourcing for High-Quality Gold Standard Development in Clinical Natural Language Processing
Source: J Med Internet Res. 2013 Apr 2;15(4):e73. doi: 10.2196/jmir.2426 (PMC3636329; doi:10.2196/jmir.2426)
Supplement: Supplementary file 3 [file jmir_v15i4e73_app3.pdf]

Definitions of Precision, Recall and F-measure

$$Precision = \frac{\#\ of\ entities\ correctly\ predicted}{\#\ of\ entities\ predicted}$$

$$Recall = \frac{\#\ of\ entities\ correctly\ predicted}{\#\ of\ positive\ entities}$$

$$F\text{-}measure = \frac{2 * Precision * Recall}{Precision + Recall}$$
