# Supplementary material for: Web 2.0-Based Crowdsourcing for High-Quality Gold Standard Development in Clinical Natural Language Processing
Source: J Med Internet Res. 2013 Apr 2;15(4):e73. doi: 10.2196/jmir.2426 (PMC3636329; doi:10.2196/jmir.2426)
Supplement: Supplementary file 4 [file jmir_v15i4e73_app4.pdf]

Voting Method Equations

$$f_{SIMPLE}(entity) = \frac{e}{j} \qquad (1)$$

$$f_{TRUST}(entity) = \frac{sum(t)}{j} \qquad (2)$$

$$f_{EXPERIENCE}(entity) = \frac{sum(\log_{10}(u) / \log_{10}(m))}{j} \qquad (3)$$
